# Context-Driven Active Contour (CDAC): A Novel Medical Image Segmentation Method Based on Active Contour and Contextual Understanding

**DOI:** 10.3390/s25092864

**Published:** 2025-04-30

**Authors:** Suane Pires Pinheiro da Silva, Roberto Fernandes Ivo, Calleo Belo Barroso, João Carlos Nepomuceno Fernandes, Thiago Ferreira Portela, Aldísio Gonçalves Medeiros, Pedro Henrique F. de Sousa, Houbing Song, Pedro Pedrosa Rebouças Filho

**Affiliations:** 1 Department of Teleinformatics Engineering, Federal University of Ceará (UFC), Fortaleza 60440-900, CE, Brazil; suanepires@lapisco.ifce.edu.br (S.P.P.d.S.); robertoivo@lapisco.ifce.edu.br (R.F.I.); 2 Federal Institute of Education, Science and Technology of Ceara (IFCE), Fortaleza 60040-531, CE, Brazil; calleo.barroso@lapisco.ifce.edu.br (C.B.B.); joao.carlos@lapisco.ifce.edu.br (J.C.N.F.); thiago.portela@lapisco.ifce.edu.br (T.F.P.); 3 Anita’s Gardens Campus, Federal University of Ceará (UFC), Itapajé 62600-000, CE, Brazil; aldisio.medeiros@lapisco.ifce.edu.br (A.G.M.); pedrofeijo@lapisco.ifce.edu.br (P.H.F.d.S.); 4 Department of Information Systems, University of Maryland, Baltimore County (UMBC), Baltimore, MD 21250, USA; songh@umbc.edu

**Keywords:** lung segmentation, computed tomography, active contour models, contextual segmentation, chronic obstructive pulmonary disease, pulmonary fibrosis, medical image processing, computer-aided diagnosis, image analysis

## Abstract

Lung diseases, including chronic obstructive pulmonary disease (COPD) and pulmonary fibrosis, pose significant health challenges due to their high morbidity and mortality rates. Computed tomography (CT) scans play a critical role in early diagnosis and disease management, yet traditional segmentation methods often falter in addressing anatomical variability and pathological complexity. To overcome these limitations, this study introduces the context-driven active contour (CDAC), a new segmentation method that combines active contour models (ACMs) with contextual analysis. CDAC leverages contextual information from image embeddings and expert annotations to refine segmentation precision. The algorithm employs contextual attention force (CAF) as an external energy term and contextual balloon force (CBF) as an internal energy term, enabling robust contour adaptation. Evaluations were conducted on CT images of healthy lungs, as well as those affected by COPD and pulmonary fibrosis. CDAC achieved notable performance metrics, including a Dice coefficient of 96.8% for healthy lungs, an Accuracy of 94.5% for COPD, and a Jaccard Index of 92.3% for pulmonary fibrosis. These results demonstrate the method’s effectiveness and adaptability. By integrating contextual insights, CDAC offers a promising solution for enhancing computer-aided diagnostic (CAD) systems in the management of lung diseases.

## 1. Introduction

Lung diseases, including chronic obstructive pulmonary disease (COPD) and pulmonary fibrosis, are among the leading causes of morbidity and mortality worldwide [1,2,3,4]. These conditions pose significant public health challenges, particularly as the global burden of respiratory diseases continues to rise due to an aging population and increasing exposure to environmental risk factors such as air pollution and tobacco use [5,6]. Early detection and accurate monitoring of these diseases are essential to improving patient outcomes, highlighting the importance of developing advanced diagnostic tools [7,8].

Computed tomography (CT) is a critical imaging modality for the diagnosis of lung diseases, providing detailed cross-sectional images that allow accurate visualization of lung structures [9]. However, traditional methods for analyzing CT images, such as manual segmentation, are time-consuming, prone to variability, and inadequate for handling complex cases with significant anatomical or pathological variability [10]. These limitations have driven the exploration of automated segmentation techniques to increase diagnostic accuracy and efficiency.

Current research in automated segmentation has made significant progress with the development of advanced methodologies, including active contour models (ACMs), region-growing algorithms, and deep learning approaches [11,12]. ACMs, introduced by [11], have been widely used for medical image segmentation due to their ability to dynamically refine contours based on energy minimization. Variants such as gradient vector flow (GVF) [13] and vector field convolution (VFC) [14] have improved the ability of ACMs to capture complex structures by extending their external force fields. Furthermore, the incorporation of fuzzy logic, as demonstrated in the edge detection approach based on general type 2 fuzzy sets (GT2 FSs) [15], explored the Lab, HSV, and RGB color spaces, enhancing robustness in color image processing. Techniques such as morphological geodesic active contour (FGAC) [16] have demonstrated effectiveness in segmenting lung regions, particularly when combined with adaptive energy terms. However, these methods often require careful initialization and parameter tuning, limiting their generalizability across diverse datasets.

ACM has been widely used for medical image segmentation, and the approach proposed in [17] reformulates the problem in terms of an energy functional within the calculus of variations, aiming to improve segmentation by handling images with non-uniform brightness. Compared to traditional approaches, this formulation seeks to make the contour more adaptable and reduce the need for reparameterization.

Region-based segmentation methods, among the earliest in medical image processing, rely on pixel intensity similarities to delineate regions of interest. Thresholding, which separates structures based on a predefined intensity, is widely used in lung segmentation but struggles with variations due to pathology or artifacts [18]. Region growing improves accuracy by iteratively expanding from a seed point but is highly sensitive to noise and requires precise initialization [19]. Level set methods offer a more flexible segmentation approach by evolving contours over time based on geometric constraints, making them effective for handling complex structures [20]. However, they can be computationally expensive and sensitive to initialization.

Deep learning-based segmentation methods have significantly advanced the field by leveraging convolutional neural networks (CNNs) to automatically extract hierarchical features from medical images. Architectures such as U-Net [21], Mask R-CNN [22], and DeepLabV3+ [23] have demonstrated excellent performance in lung segmentation tasks. However, despite their strong performance, deep learning models often lack interpretability and generalizability, particularly when trained on limited or biased datasets, and struggle to integrate contextual information effectively [24]. Still in the context of deep learning, Sharp U-Net [25] applies depthwise convolutions and a sharpening filter to adjust the feature fusion process in biomedical image segmentation. Compared to the conventional U-Net, it introduces structural modifications that influence computational efficiency and segmentation outcomes in certain applications.

Although these methodologies have advanced the field, challenges remain in balancing computational efficiency, segmentation accuracy, and adaptability. In this context, the context-driven active contour (CDAC) method is introduced as a novel solution for lung segmentation in CT images. CDAC aims to address these limitations by combining the robustness of ACMs with contextual analysis. By leveraging contextual attention mechanisms and expert annotations, CDAC increases segmentation accuracy and adapts to heterogeneous clinical scenarios, providing a promising solution for improving the diagnosis and treatment of lung diseases and making it a robust tool for computer-aided diagnosis (CAD) systems.

The main objective of this study is to demonstrate the effectiveness of CDAC in lung segmentation on CT images, particularly in cases involving complex anatomical and pathological variations. The experimental results validate the method’s performance, achieving satisfactory values compared to recent techniques. These findings highlight the potential of CDAC to advance diagnostic workflows, ultimately contributing to improved clinical decision-making and patient outcomes.

### Paper Organization

The remainder of this paper is organized as follows: Section 2 provides a detailed overview of the materials and methods employed in the development of CDAC, including dataset characteristics, the mathematical formulation of the model, and the evaluation criteria used for performance assessment. Section 3 presents the experimental results, offering a comparative analysis of CDAC against existing segmentation techniques. This section highlights the strengths and limitations of the proposed approach while discussing its applicability in medical imaging. Additionally, it includes an in-depth discussion on computational efficiency and practical deployment considerations. Section 4 summarizes the key findings of the study, addressing its limitations and outlining future research directions to enhance CDAC’s performance and extend its application to broader medical imaging domains and other fields.

## 2. Materials and Methods

This section details the methods and procedures adopted for the development and validation of context-driven active contour (CDAC). CDAC is an image segmentation method that combines the robustness of ACMs with the advanced contextual understanding capabilities provided by modern deep learning techniques originally focused on natural language processing (NLP) and adapted for image processing, with a focus on computer vision. The methodology to be described covers everything from data collection and organization, through model development, to procedures for evaluating and validating CDAC.

### 2.1. Chest CT Dataset

For the analysis of the proposed method, we used a private dataset of chest CT images, which can be accessed at this link. This dataset was built with the assistance of experts in the field to ensure high-quality annotations and reliable ground truth segmentation masks for validation. The research conducted with these data contributes new information and results, increasing the originality and scientific relevance of the study. In addition, using private data allows the inclusion of a greater diversity of clinical cases and complex conditions, which are often not sufficiently represented in public datasets. This factor strengthens the credibility of the research and expands its applicability both in the scientific context and in medical practice.

The dataset presents 36 lung CT images, acquired in collaboration with the Walter Cantídio Hospital, Federal University of Ceará (UFC), Brazil, using a GE MEDICAL SYSTEM optima CT660 specification CT scanner. This dataset consists of images in digital imaging and communications in medicine (DICOM) format, each with a resolution of 512 × 512 pixels and a pixel intensity of 16 bits. The images were manually segmented by an expert for the method analysis. The images were acquired from 12 healthy patients, 12 patients with fibrosis, and 12 with COPD.

**Figure 1:**
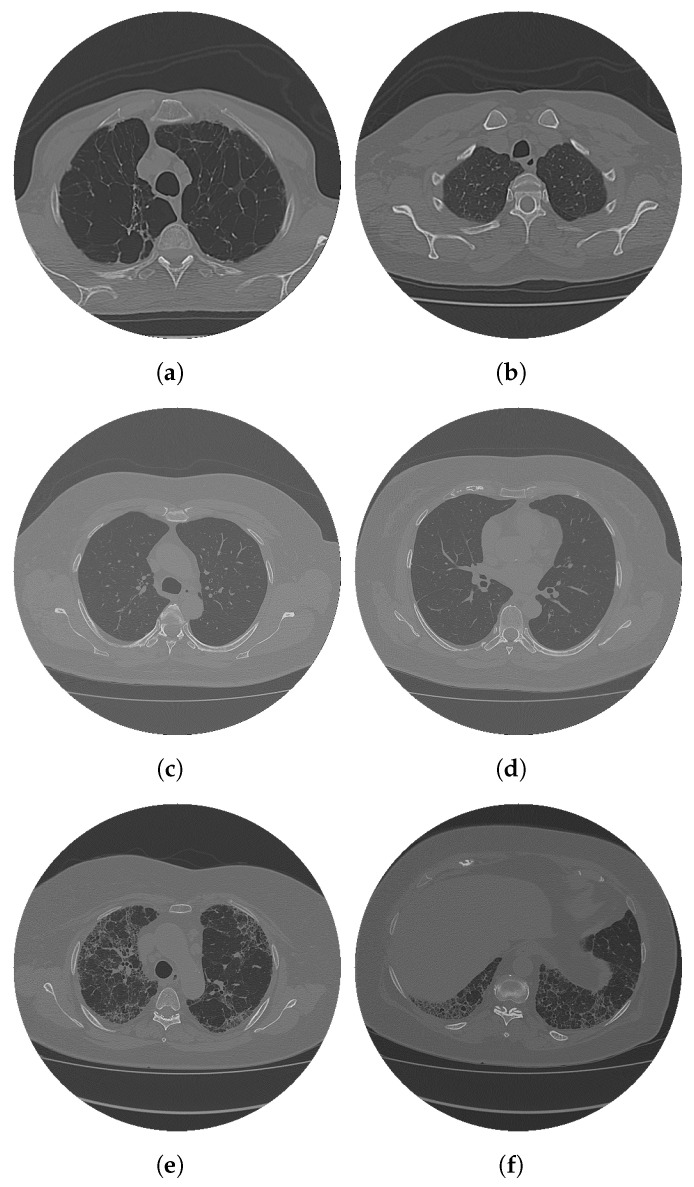
Samples from the chest CT image dataset for lung segmentation. (**a**) Sample #1 and (**b**) Sample #4 represent lungs with COPD. (**c**) Sample #16 and (**d**) Sample #21 represent healthy lungs. (**e**) Sample #30 and (**f**) Sample #36 show lungs with fibrosis.

To illustrate the diversity of this dataset and highlight its main characteristics, Figure 1 presents six representative samples. The selected images demonstrate the variation in size, shape, intensity, and contrast, highlighting the challenges inherent in the segmentation and analysis of this dataset.

Analyzing Figure 1, we can see that each image presents distinct characteristics, a fact that directly impacts the segmentation process. Some images exhibit significant variations in the size and shape of the structures, while others present significant differences in the intensity and contrast of the pixels. These variations reinforce the importance of an efficient and adaptable method, capable of dealing with different anatomical patterns and levels of complexity present in the dataset.

### 2.2. *Context-Driven Active Contour* (CDAC)

In the literature, several methodologies have been developed for the segmentation of regions of interest in medical images. Despite the variety of existing options, there is still potential to improve and enhance the reliability of these methods.

**Figure 2:**
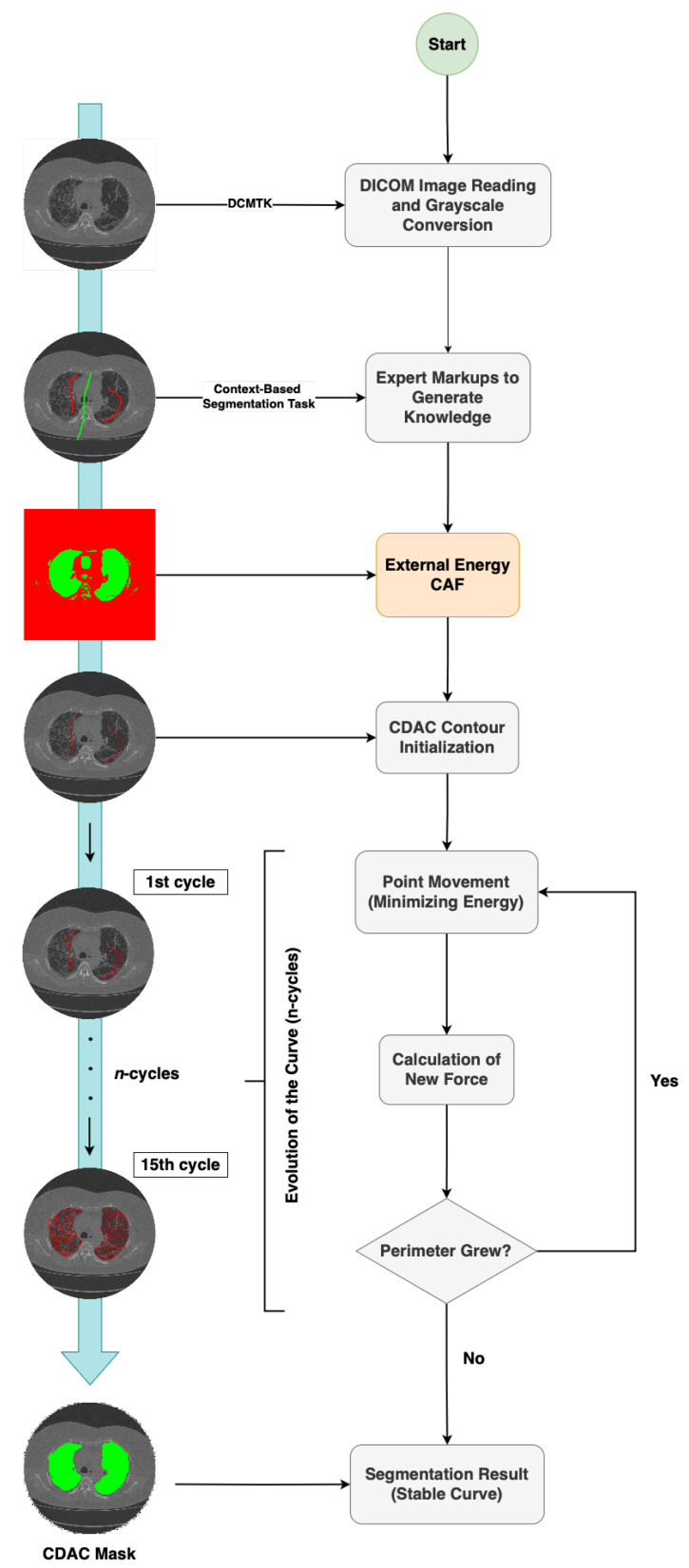
CDAC model execution pipeline.

With the intention of contributing to this field, this work presents a new technique for the segmentation of regions of interest in medical images, called context-driven active contour (CDAC). The proposed model is an image segmentation methodology that combines ACM with contextual information produced from the visual features in the image. To optimize the segmentation task, CDAC incorporates the context analysis-based processing of the Vision Transformer (ViT) [26] and proposes a new source of external energy and internal energy. Figure 2 demonstrates the pipeline that makes up the segmentation process performed by CDAC.

As we can see in Figure 2, the first step is to read the CT images, which are in DICOM format. To open images with this type of extension, the open-source DICOM toolkit (DCMTK) library provided by OFFIS is used. This tool is compatible with the C++ language, the same one used in the development of the model. After reading the image in DICOM format, it is converted to grayscale, reducing computational complexity. Since grayscale images have only one intensity channel, this transformation optimizes the algorithm’s processing, making the analysis more efficient without compromising the quality of the information relevant to segmentation.

In the next step, the expert defines the parameters of the desired context by marking two lines on the image. The first line consists of a set of points that represent the region of interest to be segmented, while the second line delimits the background region, characterizing the unwanted context. In other words, as we can see in Figure 2, the green markings indicate the region of interest, while the red markings represent the area to be ignored. We call this procedure the context-based segmentation task, which aims to transmit the expert’s technical know-how to the CDAC method, incorporating additional contextual information that a fully automated model may not be able to fully capture.

Continuing the progress presented in Figure 2, now that we have the marking curves and the grayscale image, the external energy of the proposed model, called contextual attention force (CAF), comes into play to guide the CDAC segmentation process. The external CAF energy is detailed in Section 2.2.1. With the context of interest defined and the external CAF energy calculated, the interaction between the internal and external energies of the CDAC model begins. This process deforms the segmentation curve in a controlled manner, directing its growth toward the region of interest that corresponds to the indicated context.

CDAC proposes the deformation of the curve by combining the new external energy CAF with the proposed internal energy contextual balloon force (CBF), an optimization of the internal energy presented in [12]. The internal energy CBF contributes to the coherence between the points of the curve, avoiding the formation of sharp sections or abrupt protrusions. On the other hand, the new external energy CAF attracts the points of the curve in the direction of the expansion of the contour, deforming the boundaries of the curve in the direction of the edge where the object of interest is. This deformation is governed by the minimization of the function that describes the contour.

Therefore, the external energy CAF is responsible for indicating the context of the image, mapping regions of high and low intensity. During the segmentation process, the active contour follows a greedy scheme of minimization of the energy function, in which the points of the curve are displaced towards the region of interest, characterized by lower energy. This movement continues until minimization becomes unfeasible, reducing the sum of the internal and external energies at each point. In this way, the method guarantees the reduction in the total energy of the curve, respecting the energy minimization principle of ACMs.

In the flowchart shown in Figure 2, this sequence of evolution of the curve(s) is called cycles, with the letter *n* representing the order of the results obtained in each cycle *n*. The curve is considered stable when its perimeter stops increasing after two consecutive iterations. Finally, at this point, the segmentation of the region of interest (lung) is completed, and the polygon generated by the curve, represented in green in the figure, is defined as the final result of the segmentation of the proposed method (CDAC mask) for the area of interest.

#### 2.2.1. External Energy Contextual Attention Force (CAF)

From the perspective of traditional ACM [11], which evolves the contour curve deformation as energy is minimized, the CDAC method proposes the calculation of the external energy CAF based on the image context processing, so that the energies calculated in the context reflect the regions of interest as opposed to the background regions in the image.

Let a matrix *I* represent an image, or a slice of a CT scan, whose dimensions are M×N, where *M* is the number of rows, which represents the height of the image, and *N* is the number of columns, which represents the width of the image. Each element $I_{ij}$ of the matrix represents the intensity of the pixel at position (i,j). Thus, consider the property *P*, which determines whether a point *p* in the image belongs to the low-energy region in the scan, represented by the set δ. Let p(i,j) be the pixel at position (i,j) in the image, where *i* and *j* are spatial coordinates, the property *P* can be defined as:

(1) P(p(i,j))⇔f(i,j)∈δ

where the property P(p(i,j)) is true if and only if the pixel p(i,j) belongs to the low-energy region δ, that is, when f(i,j)∈δ. Conversely, it is false if and only if the pixel p(i,j) belongs to the high-energy region Δ, that is, when f(i,j)∈Δ, so that:

(2) P(p(i,j))⇔f(i,j)∈Δ

The function f(i,j), therefore, acts as a point classification function, assigning an energy according to the context of the region in the image, being able to assign to the point (i,j) a real value that indicates the energy at the point *p*. Formally, we can write the property *P* as:

(3) $$P\left(p\left(i,j\right)\right)=\left\{\begin{array}{cc} \mathrm{True} & \mathrm{if}f(i,j)\in \delta \\ \mathrm{False} & \mathrm{if}f(i,j)\in \Delta \end{array}\right.$$

However, the previous definition presents a rigid binary classification property, not allowing adaptation regarding the calculated energy, nor delimiting the boundary between low and high energy. In this sense, we expand the previous property based on the energy threshold Λ, where Λ is a non-negative rational number. Thus, the energy of the point p(i,j) can vary in the interval [0,100]. Accordingly, let E(p(i,j)) be the energy of the point p(i,j) in the image, where E(p(i,j))∈[0,100], the property *P* can be defined in terms of the threshold Λ as follows:

(4) P(p(i,j))⇔E(p(i,j))−Λ≤0

Or, equivalently:

(5) $$P\left(p\left(i,j\right)\right)=\left\{\begin{array}{cc} \mathrm{True} & \mathrm{if}E(p(i,j))\leq \Lambda \\ \mathrm{False} & \mathrm{if}E(p(i,j))>\Lambda \end{array}\right.$$

**Figure 3:**
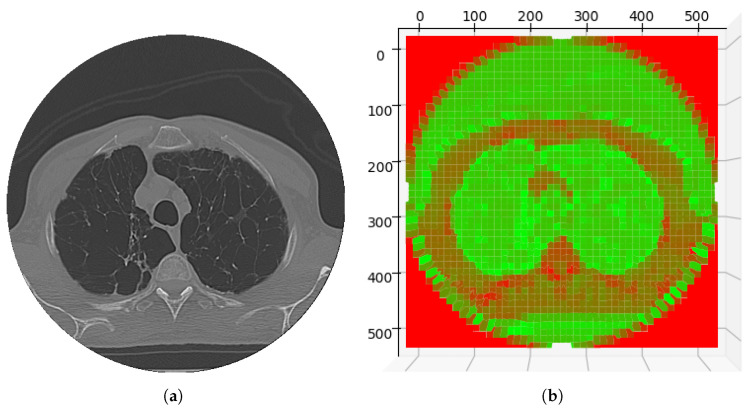
CAF energy map for sample lung CT image. (**a**) Input image. (**b**) External energy CAF.

Therefore, P(p(i,j)) is true if and only if the energy of the point p(i,j) is less than or equal to the threshold Λ, indicating that there is a low energy region. E(p(i,j)) is the function that returns the energy of the point p(i,j). The ViT algorithm processes the image context to differentiate regions of interest from background regions by analyzing energy intensity variations. In the CAF energy map shown in Figure 3, low-intensity energy values correspond to regions of interest, highlighted in green, representing structures relevant to the segmentation process. Conversely, high-intensity energy values, shown in red in Figure 3, indicate background areas or non-relevant regions.

Consider, therefore, that the ViT algorithm produces a matrix, which, within the CDAC approach, we call the context matrix C(x,y), which has dimensions equal to the original image, where each element of the matrix represents an energy value for the corresponding pixel in the image. In this sense, the point of the context matrix C(x,y) indicates low energy values when in regions of interest, that is, when C(x,y)∈δ for all C(x,y)≤Λ. On the other hand, C(x,y) assumes high values, indicating high energy when in regions of no interest, that is, when C(x,y)∈Δ for all C(x,y)>Λ. At this point, we can redefine Equation (5) in terms of the energy matrix *C*, resulting in Equation (6).

(6) $$CAF\left(p\left(i,j\right)\right)=\left\{\begin{array}{cc} \mathrm{Low}\mathrm{Energy} & \;\mathrm{if}\;C(i,j)\leq \Lambda \\ \mathrm{High}\mathrm{Energy} & \;\mathrm{if}\;C(i,j)>\Lambda \end{array}\right.$$

From Equation (6), it can be seen that the threshold Λ acts as a central parameter for the interpretation of the context matrix C(x,y). This matrix, generated based on the ViT algorithm, distributes energy values that differentiate regions of interest (C(i,j)≤Λ) from regions of no interest (C(i,j)>Λ). This distinction allows the CDAC approach to efficiently drive the active curve, focusing on areas of interest and ignoring those that do not contribute to the segmentation objective, thus optimizing the process of identifying structures of interest.

To define the external energy $E_{\mathrm{CAF}}$ in terms of the context matrix C(x,y), we can formulate the external energy as an integral over the curve v(s). Such an external energy is then proposed as:

(7) $$E_{\mathrm{CAF}}=\int_{0}^{1}C\left(v\left(s\right)\right)\;ds$$

where v(s)=[x(s),y(s)] represents the position of the active contour parameterized by *s*. The value of C(v(s)) for a point v(s) on the curve is obtained directly from the context energy matrix *C*.

The goal of the approach is to evolve the active contour in such a way as to minimize the external energy $E_{\mathrm{CAF}}$. For regions of interest with low energy C(x,y), the curve will tend to move to these regions to minimize $E_{\mathrm{CAF}}$. Therefore, preserving the internal energy terms and replacing the term that calculates the external energy, by the energy $E_{CAF}$, defined in Equation (7), we then have the calculation of the total energy CDAC ($E_{CDAC}$) given by:

(8) $$E_{CDAC}=E_{\mathrm{int}}+E_{\mathrm{CAF}}$$

where $E_{\mathrm{int}}$ is the internal energy that controls the smoothness of the contour, while the term $E_{\mathrm{CAF}}$ represents the external energy based on the context energy matrix *C*. Substituting the terms in the traditional active contour Equation [11], we have the total energy calculated as:

(9) $$E_{CDAC}=\alpha \int_{0}^{1}{\left|\frac{\partial v(s)}{\partial s}\right|}^{2}ds+\beta \int_{0}^{1}{\left|\frac{\partial^{2}v\left(s\right)}{\partial s^{2}}\right|}^{2}ds+\int_{0}^{1}M\left(v\left(s\right)\right)\;ds$$

Therefore, to minimize the total energy $E_{CDAC}$, we can use numerical methods such as gradient descent. The equation of motion for the curve can be expressed as:

(10) $$\frac{\partial v(s)}{\partial t}=-\frac{\delta E}{\delta v(s)}$$

where $\frac{\delta E}{\delta v(s)}$ is the functional derivative of the total energy *E* with respect to the contour v(s).

To construct the initial curve used in segmentation, let $P_{1},P_{2},\ldots ,P_{n}$ be the set of non-collinear points that delimit the region of interest. These points can be connected by straight line segments, forming a closed contour that will serve as input to the model. Each segment between two consecutive points $P_{i}=\left(x_{i},y_{i}\right)$ and $P_{i+1}=\left(x_{i+1},y_{i+1}\right)$ can be described by the following parametric equation:

(11) $$\left(x\left(t\right),y\left(t\right)\right)=\left(1-t\right)\cdot \left(x_{i},y_{i}\right)+t\cdot (x_{i+1},y_{i+1}),\;t\in [0,1].$$

This representation ensures the formation of a continuous curve, which is essential for the evolution of the active contour throughout the segmentation process. In the context of CDAC, this initial curve serves as a basis for the interaction between internal and external energies, allowing the model to guide the segmentation more effectively and adaptively to the image characteristics.

As for the distance function, we define *D* as the desired distance from the closed curve to the points $P_{i}$. We will use a function g(x) that maintains the distance *D* from the contoured curve to the points $P_{i}$. The parametric equation of the closed curve that contours the points $P_{1},P_{2},\ldots ,P_{n}$, keeping them within an internal region defined by *D*, can be written as shown in Equation (12):

(12) $$\left(x\left(t\right),y\left(t\right)\right)=\left(1-t\right)\cdot \left(x_{n},y_{n}\right)+t\cdot \left(x_{1},y_{1}\right)+\frac{D}{\sqrt{{\left(x_{1}-x_{n}\right)}^{2}+{\left(y_{1}-y_{n}\right)}^{2}}}\cdot (y_{1}-y_{n},-\left(x_{1}-x_{n}\right)),$$

where t∈[0,1], and:

- $\left(1-t\right)\cdot \left(x_{n},y_{n}\right)+t\cdot \left(x_{1},y_{1}\right)$ represents the linear interpolation between the points $P_{n}$ and $P_{1}$, ensuring that the closed curve starts and ends at the extreme points.
- $\frac{D}{\sqrt{{\left(x_{1}-x_{n}\right)}^{2}+{\left(y_{1}-y_{n}\right)}^{2}}}\cdot \left(y_{1}-y_{n},-\left(x_{1}-x_{n}\right)\right)$ is a perpendicular vector of magnitude *D* in relation to the segment $P_{n}P_{1}$, ensuring that the curve maintains the distance *D* of the points internally.

**Figure 4:**
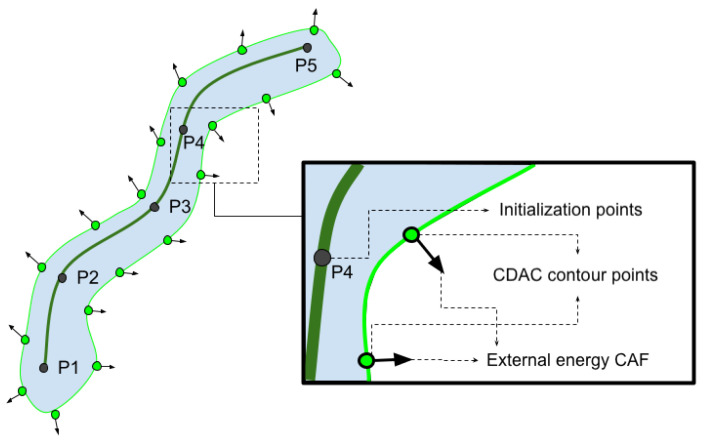
External energy CAF and the CDAC contour initialization curve.

In Figure 4, the initialization curve of the CDAC model is shown in dark green. The points $P_{n}$ to $P_{5}$ represent the initialization points. The points and the curve in light green represent the closed initialization curve of the contour, which will be deformed according to the attraction of the external energy CAF.

The interaction between the initial curve and the external energy CAF, highlighted in Figure 4, is essential for the functioning of the CDAC model. This mechanism ensures that the deformation of the curve is guided by contextual information extracted from the energy matrix C(x,y), allowing the model to maintain dynamic adaptation to the image characteristics. The arrows seen in Figure 4 indicate the direction of displacement of the points that make up the contour, allowing its evolution. In addition, the initial configuration of the points $P_{n}$ to $P_{5}$, shown as an example in this figure, has a direct impact on the efficiency and accuracy of the segmentation process, since it defines the initial proximity of the curve in relation to the object of interest.

#### 2.2.2. Internal Energy Contextual Balloon Force (CBF)

The contextual adaptive balloon internal energy represents an optimization of the adaptive internal energy proposed by [12]. This optimization has as its main characteristic to improve the segmentation process, avoiding the repetitive analysis of regions previously covered by the curve. More specifically, the proposed CDAC model considers that the regions previously analyzed by the CAF external energy were identified as part of the object of interest. Thus, the internal energy does not reevaluate these areas, focusing only on the new regions not yet segmented.

Therefore, a significant contribution of the CDAC model is the influence of the external energy CAF on the internal energy CBF, trying to optimize the speed and analysis of the curve. For example, if the external energy CAF determined that the region inside the curve corresponds to the region of the stroke or the lung, the internal energy CBF does not need to reevaluate the possibility of returning to the interior of the region of interest and, instead, must continue until it reaches the edge. Therefore, we consider the regions previously identified as the area of interest (lung) by the external energy CAF as having the maximum value of the internal energy in that area, preventing the curve from retreating to the interior of the region.

The internal energy CBF can be described mathematically in a way that incorporates the influence of the external energy CAF and optimizes segmentation, avoiding the reevaluation of previously analyzed regions. Mathematically, the internal energy CBF can be represented considering two main components: continuity and contextual adaptivity, as shown in Equation (13):

(13) $$E_{\mathrm{CBF}}\left[c\left(s\right)\right]=w_{\mathrm{cont}}\cdot F_{\mathrm{cont}}\left[c\left(s\right)\right]+w_{\mathrm{context}}\cdot F_{\mathrm{context}}\left[c\left(s\right)\right]$$

where

- $E_{\mathrm{CBF}}\left[c\left(s\right)\right]$ is the CBF internal energy along the curve c(s).
- $F_{\mathrm{cont}}\left[c\left(s\right)\right]$ is the continuity force, which maintains the smoothness of the curve.
- $F_{\mathrm{context}}\left[c\left(s\right)\right]$ is the adaptive contextual force, which adapts the force based on the contextual analysis of the region.
- $w_{\mathrm{cont}}$ and $w_{\mathrm{context}}$ are weights that adjust the influence of each term.

The continuity force $F_{\mathrm{cont}}\left[c\left(s\right)\right]$ can be expressed as:

(14) $$F_{\mathrm{cont}}\left[c\left(s\right)\right]=\alpha {\left|\frac{d}{ds}c\left(s\right)\right|}^{2}$$

where

- α is a weight that controls the stiffness of the curve.
- $\frac{d}{ds}c\left(s\right)$ is the derivative of the curve with respect to the parameter *s*, representing the continuity of the curve.

The adaptive contextual force $F_{\mathrm{context}}\left[c\left(s\right)\right]$ considers the regions previously analyzed by the external energy *CAF*:

(15) $$F_{\mathrm{context}}\left[c\left(s\right)\right]=\beta \cdot \phi \left(c\left(s\right)\right)$$

where

- β is a weight that adjusts for the influence of contextual force.
- ϕ(c(s)) is a modified step function that takes into account the influence of external energy *CAF*:
  (16) $$\phi \left(c\left(s\right)\right)=\left\{\begin{array}{cc} 1 & \mathrm{if}c(s)\mathrm{belongs}\mathrm{to}a\mathrm{region}\mathrm{previously}\mathrm{analyzed}\mathrm{as}\\ \; & \mathrm{part}\mathrm{of}\mathrm{the}\mathrm{object}\mathrm{of}\mathrm{interest};\\ 0 & \mathrm{otherwise} \end{array}\right.$$

The combination of these forces results in the internal energy CBF, as shown in Equation (17), which adapts the segmentation based on the context analyzed by the external energy CAF.

(17) $$E_{\mathrm{CBF}}\left[c\left(s\right)\right]=w_{\mathrm{cont}}\cdot \alpha {\left|\frac{d}{ds}c\left(s\right)\right|}^{2}+w_{\mathrm{context}}\cdot \beta \cdot \phi \left(c\left(s\right)\right)$$

Finally, the total energy, presented in Equation (18), which guides the evolution of the curve c(s) is the sum of the internal energy CBF and the external energy CAF:

(18) $$E_{{\mathrm{total}}_{CDAC}}\left[c\left(s\right)\right]=E_{\mathrm{CBF}}\left[c\left(s\right)\right]+E_{\mathrm{CAF}}\left[c\left(s\right)\right]$$

where

- $E_{\mathrm{CAF}}\left[c\left(s\right)\right]$ is the contextual external energy that attracts the curve to the edges of the object of interest.

Mathematically, the CBF internal energy is designed to optimize segmentation by avoiding the reanalysis of regions already identified as part of the object of interest by the external energy CAF. By focusing on new regions and combining continuity with contextual adaptability, the CBF internal energy seeks a more assertive and efficient segmentation.

### 2.3. Evaluation Metrics for Segmentation

The evaluation of the proposed method is performed using metrics widely used in the literature to measure the accuracy and effectiveness of segmentation. These metrics quantify the correspondence between the segmentation generated by the algorithm and the reference annotated by experts (ground truth), using the confusion matrix to classify the pixels into true positives (TPs), false positives (FPs), true negatives (TNs) and false negatives (FNs) [27].

In an image containing only two regions, segmentation can be interpreted as a classification process [28], where each pixel is designated as either 0 (zero) or 1 (one). In this case, 1 (one) represents that the pixel belongs to the region of interest (ROI) and 0 (zero) indicates that the pixel is part of the background region. The region of interest is called the positive class, while the background region is referred to as the negative class.

We consider quantitative metrics such as accuracy, precision, sensitivity, and specificity [29]. In the segmentation task, accuracy measures the total proportion of correctly classified pixels, while precision evaluates the proportion of correctly identified pixels in the segmented region. Sensitivity verifies the ability of the method to correctly detect the region of interest, and specificity measures the rate of pixels correctly classified as belonging to the background. In addition, the Matthews correlation coefficient (MCC) is employed to provide a balanced view of performance, especially in imbalanced datasets.

Regarding the similarity metrics employed, we considered the Jaccard index and the Dice coefficient, which aim to evaluate the overlap between the segmentation mask returned by the proposed model and the reference mask (ground truth), while the Hausdorff distance measures the largest discrepancy between the segmented contours [29]. All these metrics ensure a comprehensive analysis of the segmentation quality, considering both the spatial accuracy and the geometric fidelity of the contours generated by CDAC.

## 3. Results and Discussion

This section presents the results obtained by CDAC in lung segmentation in chest CT images. The analysis is conducted using the evaluation metrics described in Section 2, ensuring a quantitative interpretation of the performance achieved. The comparison of the CDAC results was performed with approaches that used the same data set, as cited in Section 1, ensuring consistency and scientific validity. This decision avoids potential biases caused by variations in the data and allows the observed differences to be directly attributed to the methodologies employed.

To evaluate the performance of CDAC in lung segmentation, three main perspectives were considered: (i) analysis of individual lung segmentation, (ii) analysis of general lung segmentation, and (iii) analysis of lung segmentation by comorbidities.

Individual lung analysis allows us to explore the challenges associated with the physiological and anatomical differences between the right and left lungs. Overall lung analysis provides a comprehensive view of the method’s performance, facilitating comparisons with other techniques and assessing its overall efficiency. Finally, segmented analysis by comorbidities is essential to understand how the method behaves in healthy and diseased lungs, allowing us to assess its robustness in different clinical contexts.

### 3.1. CDAC Settings

CDAC uses different ViT variants to provide contextual information that guides the segmentation process, resulting in three different configurations for the proposed model, called CDAC-A (*Advanced*), CDAC-S (*Standard*) and CDAC-X (*Extra-Advanced*). The ViT variants differ mainly in the number of layers, attention mechanism properties, *embeddings* size, and total number of parameters. The three CDAC configurations are detailed in Table 1.

Overall, CDAC-S incorporates the base variant of ViT, which is optimized to efficiently provide contextual information while balancing model complexity and generalization capability. CDAC-A utilizes the advanced variant of ViT, which improves the ability to capture complex and detailed patterns in visual data. While CDAC-X integrates the largest variant of ViT, it provides a high ability to capture complex patterns in large visual datasets, enabling more detailed and accurate analysis.

Applying the three CDAC variants to the same dataset allows a detailed comparative evaluation of performance, robustness, and computational cost of each configuration, contributing to a better understanding of how each model behaves in different conditions and with different image complexities, creating a useful benchmark for future applications.

### 3.2. Individual Analysis Approach

Segmenting lungs on CT images can present distinct challenges for the left lung and the right lung, due to their anatomical differences and proximity to other structures. Here are some things to consider when it comes to the differences between each lung:

- Anatomical complexity:
  – – Right lung: With three lobes, the right lung has a more complex anatomy, which can make segmentation more challenging. The horizontal and oblique fissures must be correctly identified to separate the lobes.
  – Left lung: With only two lobes, segmentation can be simpler due to less anatomical complexity. The presence of only one oblique fissure makes it easier to separate the lobes.
- Interference from neighboring structures:
  – Right lung: Less interference from the heart, which can simplify segmentation. However, the azygos vein and other structures can still create challenges.
  – Left lung: Proximity to the heart and deep cardiac impression can make accurate segmentation difficult, especially in the area close to the heart.
- Size and Shape:
  – Right lung: Larger volume and more lobes may provide more data for the segmentation algorithm, but they also increase complexity.
  – Left lung: Smaller volume and fewer lobes may simplify segmentation, but the shape may be more irregular due to the cardiac impression.
- Aspiration cases and anomalies:
  – Right lung: More prone to foreign body aspiration due to the more vertical bronchus, which may introduce variability in the images.
  – Left lung: Less prone to foreign body aspiration, potentially resulting in less variability.

Therefore, lung segmentation on CT images may be more challenging for the right lung due to its greater anatomical complexity, with three lobes and two fissures, compared to the left lung, which has only two lobes and one fissure. The proximity of the heart may make segmentation of the left lung more difficult, while the right lung, despite less cardiac interference, still faces challenges due to the presence of the azygos vein and other structures. The larger volume and more lobes of the right lung may increase the complexity of segmentation, while the smaller volume and irregular shape of the left lung due to the cardiac impression may simplify or complicate the process. In addition, the right lung is more prone to foreign body aspiration due to its more vertical bronchus, which may result in greater variability in images.

#### 3.2.1. Analysis Between CDAC Versions—Individual Approach

Table 2 presents the results of the different CDAC configurations in the chest CT image dataset, which were evaluated using the performance metrics accuracy (Acc), precision (Prec), sensitivity (Sen), specificity (Spe), Matthews correlation coefficient (MCC), Dice similarity coefficient (Dice), Jaccard index (Jac) and Hausdorff distance (HD).

Analyzing Table 2, it can be seen that the Accuracy is quite high in all configurations, with CDAC-X presenting the best results for both the left lung (0.9951 ± 0.0027) and the right lung (0.9940 ± 0.0034). CDAC-X also demonstrates the highest accuracy for both lungs, with values of 0.9796 ± 0.0282 for the left lung and 0.9698 ± 0.0277 for the right lung, suggesting a lower false positive rate, that is, the proportion of pixels classified as lung that are actually lung. Once again, CDAC-X leads, this time in sensitivity, with 0.9724 ± 0.0235 for the left lung and 0.9685 ± 0.0236 for the right, indicating a higher rate of detection of true positives, that is, the proportion of pixels from the lung region that are correctly identified as belonging to that region. The highest specificity value is also achieved by CDAC-X, showing values of 0.9980 ± 0.0029 for the left lung and 0.9970 ± 0.0033 for the right. In segmentation, Specificity indicates the proportion of pixels not belonging to the lung area that are correctly identified, that is, the ability to detect true negatives. The Matthews coefficient is also higher for CDAC-X, with values of 0.9662 ± 0.0157 for the left lung and 0.9600 ± 0.0178 for the right, reflecting a strong and consistent correlation since MCC takes into account all classification categories (true positives, false negatives, true negatives, and false positives) to provide a balanced measure of segmentation quality.

Regarding the similarity measures, still observing Table 2, the Dice coefficient is higher for CDAC-X, with values of 0.9686 ± 0.0151 for the left lung and 0.9629 ± 0.0169 for the right, indicating greater precision and better correspondence between the areas segmented by the method and the ground truth. The Jaccard index is also higher for CDAC-X, with 0.9396 ± 0.0279 for the left lung and 0.9290 ± 0.0308 for the right, corroborating the good precision of the segmentation. Being a more conservative measure of similarity and precision of the segmentation, higher values of the Jaccard index indicate a better correspondence between the segmented area returned by the method and the ground truth. The Hausdorff distance, which measures the greatest distance from a point on the predicted segmentation contour to the closest point on the ground truth contour, is smaller in CDAC-X, with values of 4.3793 ± 1.1334 for the left lung and 6.6373 ± 2.6732 for the right, indicating a better match and a smaller error in the contour of the region segmented by the method, since HD evaluates the maximum error between the segmented contour and the ground truth contour.

Overall, as seen in Table 2, for all configurations and metrics, left lung segmentation tends to perform slightly better compared to right lung segmentation. This fact can be explained by lower anatomical complexity (fewer lobes) and lower interference from nearby structures, facilitating segmentation, as discussed at the beginning of this section.

Regarding computational cost, Table 3 shows the average convergence times for CDAC-X, CDAC-S, and CDAC-A in lung segmentation on chest CT images. The convergence times are analyzed separately for the left lung and the right lung. Observing this table, for CDAC-S, the average convergence times are 2.32 ± 1.21 s for the left lung and 1.86 ± 0.75 s for the right lung. Regarding computational cost, these results indicate that CDAC-S is the most efficient approach in segmenting both lungs, with fast and consistent convergence times. The shorter convergence time for the right lung can be attributed to a lower segmentation complexity, despite the anatomical differences between the lungs. The consistency in convergence times, reflected by the low standard deviation, suggests that CDAC-S is a reliable approach.

Still evaluating Table 3, in contrast to CDAC-S, CDAC-X presents significantly longer average convergence times and greater variability. For the left lung, the average time is 4.42 ± 4.91 s, while for the right lung it is 6.29 ± 5.55 s. The high variability in convergence times (high standard deviation) indicates that CDAC-X has lower efficiency and consistency in lung segmentation, in relation to the computational cost. This inferior performance may be the result of its greater complexity or greater sensitivity to variations in CT images, making segmentation less predictable and more dependent on the specific characteristics of each image.

Regarding CDAC-A, it can be stated from Table 3 that it shows mixed performance. For the left lung, the average convergence time is efficient, being 2.37 ± 1.25 s, comparable to CDAC-S. However, for the right lung, the average convergence time is significantly higher, being 5.38 ± 6.45 s. The high variability in the convergence time of the right lung suggests that CDAC-A may be, like CDAC-X, also more sensitive to the specific characteristics of the right lung images, resulting in less consistent segmentation. In terms of time, this mixed performance indicates that, although CDAC-A is practically as effective as CDAC-S for segmenting the left lung, it faces significant difficulties in segmenting the right lung.

**Figure 5:**
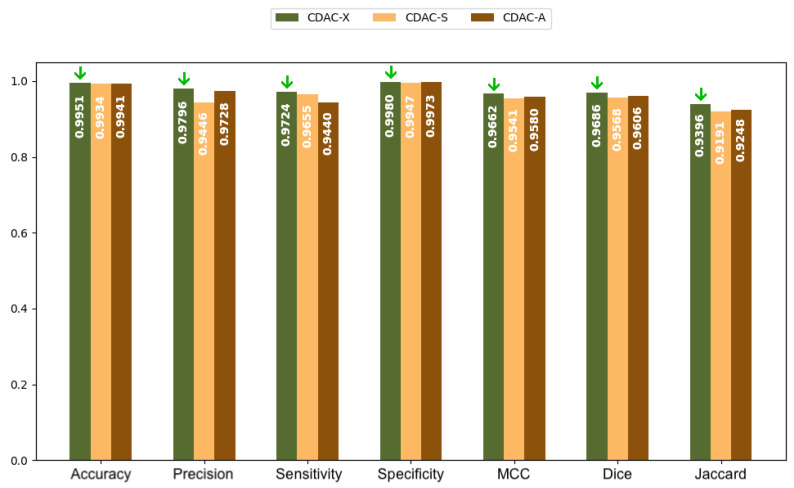
Chart for performance comparison between CDAC configurations, for segmentation of the **left lung**, based on the metrics considered.

**Figure 6:**
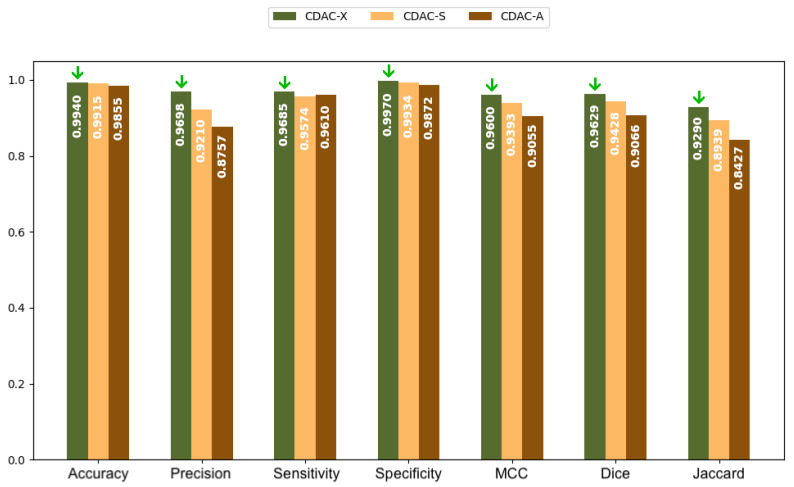
Chart for performance comparison between CDAC Configurations, for segmentation of the **right lung**, based on the metrics considered.

In Figure 5 and Figure 6, bar charts are presented, based on the values in Table 2, for both the left and right lungs, respectively. The green arrows present in all charts highlight the highest values achieved among the three CDAC configurations (CDAC-X, CDAC-S, and CDAC-A) for each metric. Their purpose is to make the interpretation of the charts more intuitive by visually emphasizing the best performance in each evaluation criterion. These charts provide a more elucidative and objective comparison of the metrics for the three CDAC configurations in lung segmentation from chest CT images dataset, considering the individual segmentation approach. Each configuration is distinguished by a specific color, with the legend included in the chart itself. The metrics are placed side by side for each configuration, allowing easy visualization of the performance of each one. The best performance for each metric is visually highlighted with a green arrow, indicating the highest value.

In summary, based on the analysis of Table 2 and Table 3, and corroborated by the graphs shown in Figure 5 and Figure 6, CDAC-X stands out as the most efficient and robust approach in lung segmentation in CT images. The green arrows highlight the best values achieved among the three CDAC configurations (CDAC-X, CDAC-S, and CDAC-A) for each metric. Their purpose is to aid in the interpretation of the graph by visually emphasizing the best performance in terms of each metric. CDAC-X not only presents the best metrics of accuracy, sensitivity, specificity and similarity metrics (Dice coefficient and Jaccard indices) for both lungs, but also demonstrates significantly shorter and more consistent convergence times, reflecting fast and reliable segmentation, compared to other versions of the method. In contrast, CDAC-S shows lower performance with longer and more variable convergence times, indicating a less optimized approach. CDAC-A, although efficient in segmenting the left lung, presents high variability and longer convergence times for the right lung, suggesting sensitivity to anatomical features and variability in the images. Thus, CDAC-X asserts itself as the most suitable solution for lung segmentation in CT images, among the other CDAC configurations.

#### 3.2.2. Comparison with Other Methods—Individual Approach

Given its superiority over other CDAC configurations, Table 4 shows a comparison of the performance of CDAC-X with other methods, considering metrics such as accuracy, precision, sensitivity, and specificity in the lung segmentation task using the dataset of chest CT images. The results are presented separately for the left and right lungs.

Analyzing Table 4, it can be noted that CDAC-X presents the best Accuracy values for both lungs, with 0.9954 ± 0.0027 for the left lung and 0.9944 ± 0.0032 for the right lung, standing out as the method with the greatest accuracy among those evaluated. Other methods, such as [12,30], also present good accuracies, but do not surpass CDAC-X. Although CDAC-X presents a high precision, with 0.9796 ± 0.0282 for the left lung and 0.9698 ± 0.0277 for the right, the method of [31] outperforms CDAC-X in this metric, with values of 0.9935 ± 0.0133 for the left lung and 0.9939 ± 0.0109 for the right. However, the high precision of [31] is accompanied by a lower sensitivity, which indicates a trade-off between false positives and false negatives. Still regarding sensitivity, CDAC-X presents the highest in this metric for both lungs, with values of 0.9777 ± 0.0157 for the left lung and 0.9691 ± 0.0165 for the right, indicating that CDAC-X is effective in correctly identifying the pixels belonging to the lung, minimizing the number of false negatives. Other methods, such as [12], also present good sensitivities, but do not reach the values of CDAC-X. Regarding specificity, the method of [31] presents the highest specificity, with values of 0.9994 ± 0.0013 for the left lung and 0.9991 ± 0.0018 for the right. CDAC-X also presents high specificity, with 0.9980 ± 0.0029 for the left lung and 0.9970 ± 0.0033 for the right, although slightly lower than that of [31].

Table 5 shows a comparison of the results of CDAC-X with other lung segmentation methods in CT images, using the performance metrics Dice coefficient, Jaccard index, Matthews correlation coefficient, and Hausdorff distance. The results are presented separately for the left and right lungs.

**Table 4:** Comparison of CDAC-X results with other works for lung dataset segmentation—individual approach (part 1).

Methods	Accuracy		Precision		Sensitivity		Specificity	
	**Left Lung**	**Right Lung**	**Left Lung**	**Right Lung**	**Left Lung**	**Right Lung**	**Left Lung**	**Right Lung**
**CDAC-X** ^ 3^	**0.9954 ± 0.0027**	**0.9944 ± 0.0032**	0.9796 ± 0.0282	0.9698 ± 0.0277	**0.9777 ± 0.0157**	**0.9691 ± 0.0165**	0.9980 ± 0.0029	0.9970 ± 0.0033
He et al. (2023) [32]	0.9835 ± 0.0075	0.9828 ± 0.0078	0.9315 ± 0.0418	0.9303 ± 0.0435	0.9321 ± 0.0420	0.9297 ± 0.0438	0.9830 ± 0.0072	0.9825 ± 0.0077
Filho et al. (2019) [31]	0.9878 ± 0.0039	0.9879 ± 0.0039	**0.9935 ± 0.0133 ^4^**	**0.9939 ± 0.0109**	0.8618 ± 0.0437	0.8640 ± 0.0510	**0.9994 ± 0.0013**	**0.9991 ± 0.0018**
Filho et al. (2014) [12]	0.9951 ± 0.0027	0.9938 ± 0.0034	0.9569 ± 0.0599	0.9620 ± 0.0307	0.9724 ± 0.0235	0.9685 ± 0.0236	0.9969 ± 0.0032	0.9965 ± 0.0031
Felix et al. (2013) [33]	0.9770 ± 0.0261	0.9806 ± 0.0194	0.8651 ± 0.1673	0.8839 ± 0.1131	0.8674 ± 0.1966	0.9096 ± 0.0938	0.9871 ± 0.0154	0.9874 ± 0.0153
Rebouças Filho et al. (2011) [34]	0.9901 ± 0.0053	0.9893 ± 0.0065	0.9783 ± 0.0561	0.9638 ± 0.0614	0.9003 ± 0.0402	0.9085 ± 0.0336	0.9985 ± 0.0036	0.9967 ± 0.0052
Alexandria et al. (2010) [35]	0.9881 ± 0.0074	0.9874 ± 0.0097	0.9354 ± 0.0904	0.9437 ± 0.0596	0.9175 ± 0.0267	0.9124 ± 0.0353	0.9942 ± 0.0073	0.9939 ± 0.0086
Felix et al. (2009) [30]	0.9948 ± 0.0022	0.9940 ± 0.0036	0.9786 ± 0.0367	0.9802 ± 0.0283	0.9528 ± 0.0256	0.9471 ± 0.0251	0.9985 ± 0.0019	0.9980 ± 0.0033
Li and Acton (2006) [14]	0.9899 ± 0.0050	0.9888 ± 0.0090	0.9293 ± 0.1487	0.9577 ± 0.0516	0.9215 ± 0.0304	0.9145 ± 0.0387	0.9961 ± 0.0048	0.9954 ± 0.0078
Xu and Prince (1998) [13]	0.9885 ± 0.0085	0.9791 ± 0.0318	0.9533 ± 0.0827	0.9658 ± 0.0507	0.9126 ± 0.0424	0.8678 ± 0.1282	0.9959 ± 0.0060	0.9958 ± 0.0093

^3^ The green background highlights instances where CDAC achieved the highest performance in a given metric, with white text ensuring readability. ^4^ Bold values indicate cases where another method outperformed CDAC in a specific metric.

**Table 5:** Comparison of CDAC-X results with other works for segmentation of the lung dataset—individual approach (part 2).

Methods	Dice		Jaccard		MCC		Hausdorff	
	**Left Lung**	**Right Lung**	**Left Lung**	**Right Lung**	**Left Lung**	**Right Lung**	**Left Lung**	**Right Lung**
**CDAC-X** ^ 5^	**0.9686 ± 0.0151**	0.9641 ± 0.0166	**0.9396 ± 0.0279**	0.9312 ± 0.0304	**0.9662 ± 0.0157**	0.9613 ± 0.0176	**4.3818 ± 1.1329**	6.6402 ± 2.6732
He et al. (2023) [32]	0.9429 ± 0.0259	0.9418 ± 0.0266	0.8930 ± 0.0459	0.8915 ± 0.0470	0.9336 ± 0.0276	0.9312 ± 0.0283	32.9000 ± 1.8200	33.1700 ± 1.8300
Filho et al. (2019) [31]	0.9222 ± 0.0235	0.9234 ± 0.0271	0.8566 ± 0.0406	0.8589 ± 0.0461	0.9186 ± 0.0209	0.9200 ± 0.0254	6.2447 ± 1.1128	7.4814 ± 2.7366
Filho et al. (2014) [12]	0.9659 ± 0.0323	**0.9651 ± 0.0149 ^6^**	0.9357 ± 0.0550	**0.9330 ± 0.0274**	0.9641 ± 0.0304	**0.9622 ± 0.0158**	4.5954 ± 1.1834	**6.0330 ± 2.6641**
Felix et al. (2013) [33]	0.8499 ± 0.1782	0.8877 ± 0.0800	0.7688 ± 0.1961	0.8063 ± 0.1140	0.8466 ± 0.1712	0.8825 ± 0.0786	17.4651 ± 2.1102	16.9211 ± 3.2122
Rebouças Filho et al. (2011) [34]	0.9358 ± 0.0291	0.9336 ± 0.0322	0.8807 ± 0.0498	0.8771 ± 0.0536	0.9323 ± 0.0280	0.9291 ± 0.0330	8.9734 ± 1.4054	11.8374 ± 2.8111
Alexandria et al. (2010) [35]	0.9229 ± 0.0497	0.9263 ± 0.0312	0.8603 ± 0.0734	0.8642 ± 0.0524	0.9181 ± 0.0460	0.9203 ± 0.0355	10.1758 ± 1.6466	12.5796 ± 1.9638
Felix et al. (2009) [30]	0.9647 ± 0.0167	0.9628 ± 0.0155	0.9323 ± 0.0304	0.9287 ± 0.0279	0.9624 ± 0.0161	0.9599 ± 0.0166	8.7083 ± 1.4481	11.0324 ± 1.8023
Li and Acton (2006) [14]	0.9161 ± 0.0999	0.9341 ± 0.0279	0.8571 ± 0.1309	0.8777 ± 0.0479	0.9152 ± 0.0861	0.9290 ± 0.0314	7.2563 ± 1.2725	9.8863 ± 1.5893
Xu and Prince (1998) [13]	0.9292 ± 0.0471	0.9065 ± 0.0843	0.8710 ± 0.0731	0.8383 ± 0.1214	0.9249 ± 0.0449	0.9015 ± 0.0858	6.4655 ± 1.1463	9.1117 ± 1.5038

^5^ The green background highlights instances where CDAC achieved the highest performance in a given metric, with white text ensuring readability. ^6^ Bold values indicate cases where another method outperformed CDAC in a specific metric.

Observing Table 5, it can be seen that, regarding the Dice coefficient, CDAC-X achieves the best results for the left lung, with 0.9686 ± 0.0151, and a satisfactory performance for the right lung, with 0.9641 ± 0.0166. The method by [12] presents a performance comparable to CDAC-X for the right lung (0.9651 ± 0.0149), but slightly inferior for the left lung. These values indicate that CDAC-X has high accuracy in segmentation, with excellent correspondence between the segmented area and the ground truth. In the Jaccard index, CDAC-X presents the best results for the left lung (0.9396 ± 0.0279) and a good performance for the right lung (0.9312 ± 0.0304). The [12] method again has a comparable performance for the right lung (0.9330 ± 0.0274), but inferior for the left lung. Regarding the Matthews coefficient, CDAC-X leads with values of 0.9662 ± 0.0157 for the left lung and 0.9613 ± 0.0176 for the right lung. The [12] method also demonstrates comparable performance for the right lung (0.9622 ± 0.0158), but inferior for the left lung. These results indicate that CDAC-X provides a balanced and accurate segmentation, taking into account all aspects of pixel classification in the lung segmentation task. Knowing that the Hausdorff distance measures the largest distance between the segmented contour points and the ground truth points, CDAC-X presents the smallest Hausdorff distance for the left lung, with 4.3818 ± 1.1700, indicating a smaller contour error. For the right lung, CDAC-X returns a distance of 6.6402 ± 1.1700, which is competitive, but slightly superior to the method by [12], which presents an HD of 6.0330 ± 1.1700. These values suggest that CDAC-X provides a segmentation with contours very close to those of the ground truth, especially for the left lung.

Therefore, in an individual lung segmentation approach, the combined analysis of Table 4 and Table 5 highlights CDAC-X as an efficient and satisfactory approach for lung segmentation in CT images, although it is not infallible. CDAC-X particularly stands out in the metrics of accuracy (0.9954 ± 0.0027 for the left lung and 0.9944 ± 0.0032 for the right lung) and sensitivity (0.9777 ± 0.0157 and 0.9691 ± 0.0165, respectively), indicating a high rate of true positive detection with a reduced number of false negatives. In the left lung, where there is less anatomical complexity due to the presence of only two lobes and the proximity of the heart, high accuracy and sensitivity indicate that the method handles cardiac interference well and can segment accurately. For the right lung, which has greater anatomical complexity with three lobes and greater volume, these results reflect the ability of CDAC-X to effectively manage this complexity.

Additionally, CDAC-X also presents the best Dice (0.9686 ± 0.0151 for the left lung) and Jaccard (0.9396 ± 0.0279 for the left lung) indices, suggesting an excellent overlap between the segmented and real areas, especially in the left lung. However, for the right lung, where the anatomical complexity is higher, other approaches, such as [12], showed comparable or slightly superior performance in some specific metrics.

Other approaches, such as those of [12,31], showed strengths in specific metrics. For example, ref. [31] achieved superior accuracy (0.9935 ± 0.0133 for the left lung and 0.9939 ± 0.0109 for the right) and exceptional specificity, indicating a very effective ability to avoid false positives. This suggests that for applications where accuracy is more critical than sensitivity, this approach may be preferred. When we consider the Hausdorff distance metric, which measures the accuracy of segmented contours, CDAC-X again proves competitive, with 6.6402 ± 1.1700, but not necessarily the best for the right lung, where [12] performs slightly better, with 6.0330 ± 2.6641.

### 3.3. General Analysis Approach

Lung segmentation in CT images is a fundamental task that is important for supporting a variety of medical applications, including lung disease diagnosis, treatment planning, and patient monitoring. In this section, we will discuss the segmentation of both lungs simultaneously, taking into account the challenges of achieving sufficient segmentation. While individual segmentation is useful in specific cases, such as anatomical anomalies or localized lesions, the general approach is most effective for medical applications that require a comprehensive and integrated view of the lung structure.

#### 3.3.1. Analysis Between CDAC Versions—General Approach

Table 6 presents the results of the performance metrics for the three CDAC configurations (CDAC-X, CDAC-S and CDAC-A) in lung segmentation using the dataset of chest CT images, adopting a general approach that considers the segmentation of both lungs simultaneously. The metrics evaluated were accuracy (Acc), precision (Prec), sensitivity (Sen), specificity (Spe), Matthews correlation coefficient (MCC), Dice similarity coefficient (Dice), Jaccard index (Jac) and Hausdorff distance (HD).

Observing Table 6, it can be seen that CDAC-X achieved the highest Accuracy value (0.9891 ± 0.0058), followed by CDAC-S (0.9849 ± 0.0090) and CDAC-A (0.9797 ± 0.0172). In this regard, the superiority of CDAC-X indicates that it is more reliable in correctly identifying pixels belonging and not belonging to the lung region compared to the other configurations. As for precision, again, CDAC-X leads with a precision of 0.9694 ± 0.0310, while CDAC-S (0.9332 ± 0.0567) and CDAC-A (0.9322 ± 0.0800) have lower performances, suggesting a better performance of CDAC-X in minimizing false positives, that is, this version returns a better proportion of pixels classified as ROI (lung) that are actually ROI.

In terms of sensitivity, in Table 6, CDAC-X presents the highest sensitivity (0.9711 ± 0.0216), indicating its superiority in detecting true positives. The CDAC-S (0.9622 ± 0.0236) and CDAC-A (0.9524 ± 0.0251) configurations have slightly lower performance, showing that CDAC-X is more effective in avoiding false negatives. Regarding specificity, CDAC-X obtained the highest specificity (0.9934 ± 0.0075), while CDAC-S (0.9867 ± 0.0111) and CDAC-A (0.9828 ± 0.0236) had lower performances, a fact that reinforces CDAC-X as the most reliable in correctly distinguishing non-lung pixels, i.e., false negatives. For the Matthews coefficient, CDAC-X achieved the highest score (0.9587 ± 0.0181), followed by CDAC-S (0.9423 ± 0.0337) and CDAC-A (0.9288 ± 0.0466), further highlighting the robustness and accuracy of CDAC-X compared to the other configurations.

Still evaluating Table 6, now observing the similarity metrics, it can be seen that CDAC-X returned the highest Dice coefficient value, with 0.9652 ± 0.0163, indicating an excellent overlap between the segmented area and the reference area (ground truth). CDAC-S (0.9506 ± 0.0317) and CDAC-A (0.9397 ± 0.0399) obtained lower values in this measure, suggesting lower precision in segmentation compared to CDAC-X. Regarding the Jaccard index, CDAC-X also leads with the highest Jaccard index, which reached 0.9332 ± 0.0297 in this metric, confirming that it achieved the best correspondence between the area segmented by the method and the reference area represented in comparison with CDAC-S (0.9075 ± 0.0539) and CDAC-A (0.8888 ± 0.0676). For the Hausdorff distance, CDAC-X presented the lowest Hausdorff distance, with 5.2922 ± 1.1140, indicating more precise contours and less segmentation error. CDAC-S and CDAC-A had lower performances, with 5.7942 ± 1.0968 and 8.2371 ± 1.1682, respectively, and, even more so, CDAC-A showed greater variability (standard deviation) and error in the segmented contours.

Table 7 shows the average convergence times for CDAC-X, CDAC-S and CDAC-A in lung segmentation on chest CT images, for general approach segmentation. Analyzing this table, it can be seen that CDAC-S demonstrates an average convergence time of 3.53 ± 1.00 s, being the shortest convergence time among the three configurations. The low standard deviation indicates significant consistency in processing times, suggesting that CDAC-S is not only fast, but also reliable in its execution.

On the other hand, still analyzing Table 7, CDAC-X presents a significantly higher average convergence time, of 7.42 ± 5.24 s. This time is more than twice the average convergence time of CDAC-S and presents a high standard deviation, indicating a large variability in processing times. This fact suggests that CDAC-X is less efficient and less predictable, and may be affected by variability in the execution conditions or in the characteristics of the CT images. As for CDAC-A, it returns an average convergence time of 5.62 ± 4.64 s, which is intermediate between CDAC-S and CDAC-X. Although the average convergence time of CDAC-A is shorter than that of CDAC-X, the standard deviation is still relatively high, indicating some instability in processing times. This suggests that although CDAC-A is more efficient than CDAC-X, it still faces challenges in terms of segmentation time consistency.

**Figure 7:**
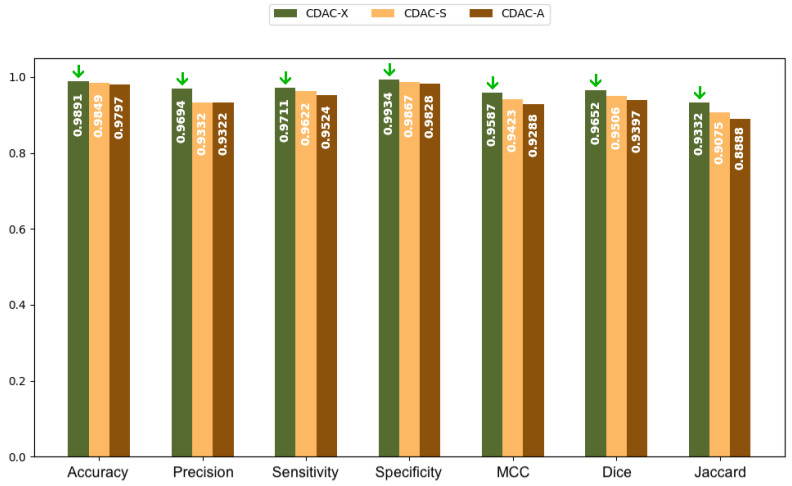
Chart for performance comparison between CDAC configurations, for lung segmentation, from the metrics presented, in a general approach.

Figure 7 shows a bar chart based on the values in Table 6. This chart provides a clearer and more direct comparison of the metrics for the three CDAC configurations in lung segmentation, using the chest CT dataset images and considering the general segmentation approach. Each configuration is represented by a distinct color, with the legend included in the chart itself. The metrics are presented side by side for each configuration, making it easy to visualize the performance of each. The best performance for each metric is visually highlighted with a green arrow indicating the highest value.

Thus, based on the analysis of Table 6 and Table 7, and confirmed by the graph shown in Figure 7, CDAC-X was not the fastest version, but it still proved to be the most efficient in terms of overall performance, among the versions of the method, considering the general approach. This configuration stood out in all metrics, showing the best accuracy (0.9891 ± 0.0058), precision (0.9694 ± 0.0310), sensitivity (0.9711 ± 0.0216), specificity (0.9934 ± 0.0075), Matthews coefficient (0.9587 ± 0.0181), Dice coefficient (0.9652 ± 0.0163) and Jaccard index (0.9332 ± 0.0297). In addition, CDAC-X presented the smallest Hausdorff distance (14.9272 ± 23.3699), indicating greater precision in the segmented contours. CDAC-X also demonstrated the lowest mean convergence time (3.53 ± 1.00 s), demonstrating its superiority in terms of accuracy and operational efficiency. In contrast, CDAC-S and CDAC-A showed greater variability and lower efficiency, with longer convergence times.

**Figure 8:**
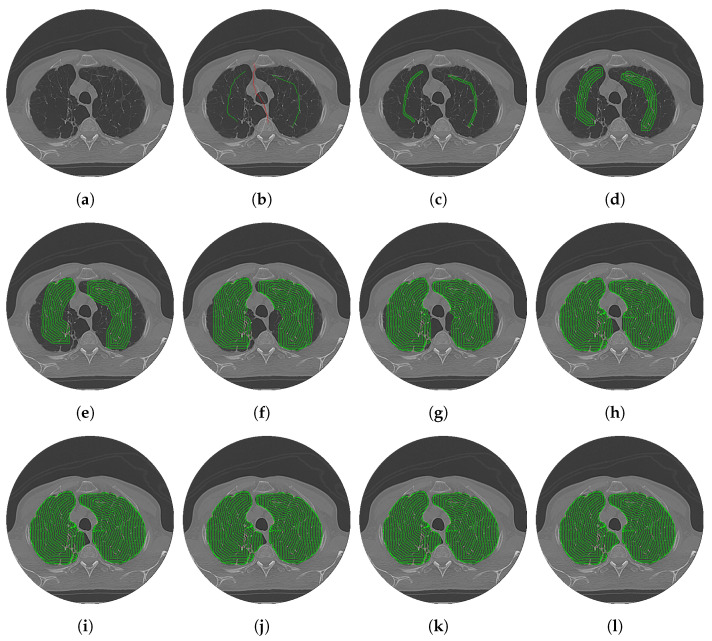
Example of CDAC-X contour evolution (in color green) for a sample of the lung segmentation dataset. (**a**) Original image, (**b**) expert markings, (**c**) 1st iteration, (**d**) 3rd iteration, (**e**) 6th iteration, (**f**) 9th iteration, (**g**) 12th iteration, (**h**) 15th iteration, (**i**) 18th iteration, (**j**) 25th iteration, (**k**) 30th iteration, and (**l**) 36th iteration (stabilized contour).

Figure 8 shows the evolution of the contour during segmentation using CDAC-X for lung. This evolution is represented in green in the figure. Figure 8a shows the original image. Figure 8b shows the markings made by the expert to guide the segmentation process of the proposed model. From Figure 8c–l, it is possible to follow the progress of the iterations, from the first to the thirty-sixth, where the contour stabilizes.

**Figure 9:**
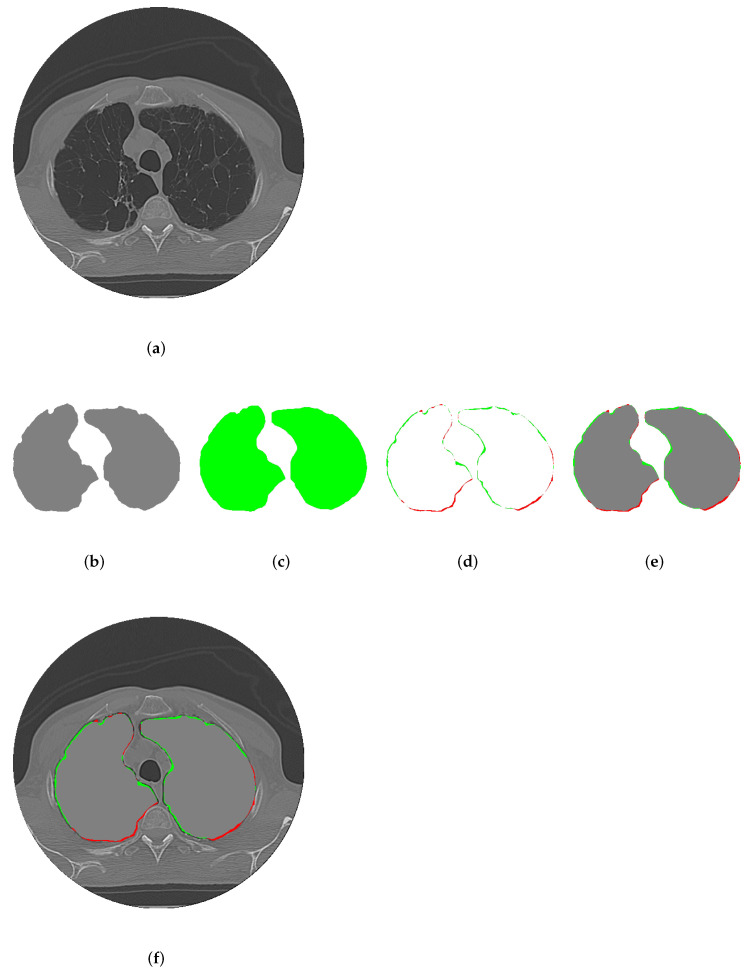
Qualitative analysis between CDAC-X segmentations and the ground truth for sample #1 of the lung dataset, presenting, visually, the differences between the prediction of the proposed method and the expert’s judgment. (**a**) Original image. (**b**) Ground truth mask. (**c**) CDAC-X segmentation mask. (**d**) FN (in red) and FP (in green). (**e**) Ground truth with (FN + FP). (**f**) Overlaying ground truth with (FN + FP) over the original image.

Figure 9 presents a qualitative analysis of the segmentations performed by the CDAC-X method in comparison with the ground truth, using a sample of the CT image dataset for lung segmentation. Figure 9a shows the original sample image. Figure 9b shows the reference mask (ground truth) for ideal segmentation. Figure 9c shows the segmentation mask generated by the CDAC-X method, in green. Figure 9d highlights the false negatives (in color red) and false positives (in color green) identified in the segmentation performed by CDAC-X in comparison with the ground truth.

Figure 9e shows the overlap of the ground truth mask with false negatives and positives, making it easier to visualize the discrepancies. Finally, Figure 9f shows these overlaps on the original image, providing a complete view of the differences between the segmentation of the proposed method and the reference segmentation performed by experts. Specifically, for the sample presented as an example in Figure 9, CDAC-X achieved accuracy, precision, Matthews coefficient, Dice coefficient, and Hausdorff distance values of 0.9987, 0.9960, 0.9578, 0.9577, and 4.2426, respectively.

#### 3.3.2. Comparison with Other Methods—General Approach

Due to its superiority compared to other CDAC configurations, Table 8 presents a comparison of the performance of CDAC-X with other methods, considering metrics such as accuracy, sensitivity and Matthews correlation coefficient (MCC) in the lung segmentation task using the chest CT images dataset, adopting the general segmentation approach.

Analyzing Table 8, it can be seen that the CDAC-X method presents an accuracy of 0.9891 ± 0.0058, standing out as one of the most accurate methods in lung segmentation. This high accuracy value indicates that CDAC-X is effective in correctly identifying both true positives and true negatives. In comparison, the [36] method slightly outperforms CDAC-X with an accuracy of 0.9897 ± 0.0065, closely followed by the [12] method with 0.9898 ± 0.0057. In terms of sensitivity, the CDAC-X method achieves 0.9711 ± 0.0216, reflecting its ability to correctly detect most areas of interest in the lung. This value is surpassed only by the method of [37], which obtains a sensitivity of 0.9921 ± 0.0066, indicating an even more effective detection of positive regions.

Other methods such as [12,38] also present high sensitivity, but do not surpass the performance of [37]. Regarding the Matthews coefficient, CDAC-X stands out significantly, with a score of 0.9637 ± 0.0181, the best among all the methods compared. The MCC is a metric that considers all elements of the confusion matrix, offering a balanced assessment of the model’s performance in imbalanced datasets. Comparatively, methods such as [12,30] also present high MCC, with 0.9612 ± 0.0183 and 0.9586 ± 0.0110, respectively, but still lag behind CDAC-X. This result indicates that CDAC-X is particularly effective in minimizing errors in all categories, providing a more balanced and reliable performance.

In Table 9, a comparison of the results of CDAC-X with other lung segmentation methods in CT images is performed, using performance metrics such as Dice coefficient, Jaccard index and Hausdorff distance. Evaluating this table, it can be observed that CDAC-X achieves a Dice score of 0.9680 ± 0.0163, standing out as the best among the compared methods. The Dice metric measures the similarity between the segmented result and the ground truth, and a higher score indicates a near-perfect overlap. In comparison, methods such as [12], with 0.9672 ± 0.0170, and [37], with 0.9619 ± 0.0193, also present good results, but are slightly behind CDAC-X. The high score of CDAC-X demonstrates its superiority in segmenting the lung region with high precision and fidelity to the ground truth.

**Table 8:** Comparison of CDAC-X results with other works in lung dataset segmentation—general approach (part 1).

Methods	Accuracy	Sensitivity	MCC
**CDAC-X** ^9^	0.9891 ± 0.0058	0.9711 ± 0.0216	**0.9637 ± 0.0181 ^9^**
He et al. (2023) [32]	0.9828 ± 0.0075	0.9309 ± 0.0429	0.9327 ± 0.0280
de S. Rebouças et al. (2021) [36]	0.9897 ± 0.0065	0.9857 ± 0.0178	0.9473 ± 0.0150
Braga et al. (2021) [39]	0.9717 ± 0.0109	0.8248 ± 0.0616	0.8854 ± 0.0367
Medeiros et al. (2020) [37]	0.9886 ± 0.0046	**0.9921 ± 0.0066 ^10^**	0.9554 ± 0.0132
Filho et al. (2019) [31]	0.9731 ± 0.0079	0.8489 ± 0.0471	0.9036 ± 0.0226
Braga et al. (2017) [38]	0.9884 ± 0.0046	0.9739 ± 0.0287	0.9555 ± 0.0138
Filho et al. (2014) [12]	**0.9898 ± 0.0057**	0.9736 ± 0.0141	0.9612 ± 0.0183
Felix et al. (2013) [33]	0.9577 ± 0.0396	0.9768 ± 0.0345	0.8538 ± 0.1185
Rebouças Filho et al. (2011) [34]	0.9803 ± 0.0088	0.9058 ± 0.0344	0.9266 ± 0.0269
Alexandria et al. (2010) [35]	0.9756 ± 0.0163	0.9159 ± 0.0287	0.9133 ± 0.0305
Felix et al. (2009) [30]	0.9888 ± 0.0052	0.9501 ± 0.0229	0.9586 ± 0.0110
Li and Acton (2006) [14]	0.9788 ± 0.0128	0.9189 ± 0.0333	0.9212 ± 0.0315
Xu and Prince (1998) [13]	0.9678 ± 0.0357	0.8868 ± 0.0791	0.8999 ± 0.0637

^9^ The green background highlights instances where CDAC achieved the highest performance in a given metric, with white text ensuring readability. ^10^ Bold values indicate cases where another method outperformed CDAC in a specific metric.

On the other hand, in the Jaccard metric, CDAC-X also stands out with a score of 0.938 ± 0.0297, again the best among all the methods evaluated. The Jaccard metric, similar to Dice, measures the similarity and diversity of the segmented samples, but in a more rigorous way. Methods such as [12], with 0.937 ± 0.0309, and [37], with 0.9273 ± 0.0343, present similar results, but none surpasses CDAC-X. The high Jaccard score of CDAC-X confirms its effectiveness in performing accurate and consistent segmentations, surpassing the other methods in terms of similarity to the ground truth. The CDAC-X method obtains a Hausdorff score of 5.2922 ± 1.1134, which is competitive, but not the best. The [36] method presents the best performance with 4.2400 ± 0.4300, followed by [12] with 5.1400 ± 1.1700.

**Table 9:** Comparison of CDAC-X results with other works in lung dataset segmentation—general approach (part 2).

Methods	Dice	Jaccard	Hausdorff
**CDAC-X** ^11^	**0.9680 ± 0.0163**	**0.9382 ± 0.0297**	5.2922 ± 1.1134
He et al. (2023) [32]	0.9424 ± 0.0263	0.8922 ± 0.0465	33.0336 ± 1.8253
de S. Rebouças et al. (2021) [36]	0.9551 ± 0.0142	0.9145 ± 0.0262	**4.2400 ± 0.4300 ^12^**
Braga et al. (2021) [39]	0.8971 ± 0.0401	0.8157 ± 0.0618	119.4400 ± 39.5800
Medeiros et al. (2020) [37]	0.9619 ± 0.0193	0.9273 ± 0.0343	5.5700 ± 1.1700
Filho et al. (2019) [31]	0.9158 ± 0.0256	0.8456 ± 0.0437	77.7383 ± 1.7590
Braga et al. (2017) [38]	0.9623 ± 0.0134	0.9276 ± 0.0245	88.8900 ± 31.4300
Filho et al. (2014) [12]	0.9672 ± 0.0170	0.9371 ± 0.0309	5.1400 ± 1.1700
Felix et al. (2013) [33]	0.8729 ± 0.1118	0.7882 ± 0.1443	19.0498 ± 1.7982
Rebouças Filho et al. (2011) [34]	0.9372 ± 0.0266	0.8830 ± 0.0449	10.7655 ± 1.2027
Alexandria et al. (2010) [35]	0.9281 ± 0.0221	0.8665 ± 0.0379	13.0924 ± 1.1400
Felix et al. (2009) [30]	0.9651 ± 0.0099	0.9329 ± 0.0183	6.6433 ± 1.1095
Li and Acton (2006) [14]	0.9333 ± 0.0278	0.8761 ± 0.0474	11.9261 ± 1.1579
Xu and Prince (1998) [13]	0.9176 ± 0.0477	0.8510 ± 0.0761	14.3020 ± 1.3013

^11^ The green background highlights instances where CDAC achieved the highest performance in a given metric, with white text ensuring readability. ^12^ Bold values indicate cases where another method outperformed CDAC in a specific metric.

With an emphasis on computational cost, Table 10 provides a comparison between CDAC-X and different methods that addressed lung segmentation in chest CT images. The CDAC-X method, with an execution time of 7.42 s, proves to be acceptable, but not the fastest. Methods such as [39], which presents an extremely low time of 0.13 s, and [36], with 1.32 s, are significantly faster, offering advantages in terms of computational cost. However, it is worth noting that the methods of [13,14] show very high execution times, 30 and 240 s, respectively, severely limiting their applicability in contexts that require speed.

**Table 10:** Comparison of the average convergence time of the CDAC-X version with other works in the segmentation of the lung dataset—general approach.

Method	Time (s)
**CDAC-X**	7.42 ± 5.24
He et al. (2023) [32]	1.15 ± 2.75
de S. Rebouças et al. (2021) [36]	1.32 ± 0.09
Braga et al. (2021) [39]	**0.13 ± 0.02 ^13^**
Medeiros et al. (2020) [37]	1.85 ± 0.50
Filho et al. (2019) [31]	5.86 ± 1.96
Braga et al. (2017) [38]	2.01 ± 0.46
Filho et al. (2014) [12]	2.00 ± 0.16
Felix et al. (2009) [30]	4.90 ± 2.02
Li and Acton (2006) [14]	30.00 ± 2.67
Xu and Prince (1998) [13]	240.00 ± 3.05

^13^ Bold values indicate cases where another method outperformed CDAC in a specific metric.

Overall, the CDAC method proves to be a satisfactory solution for lung segmentation, combining high accuracy and segmentation quality. CDAC-X presents excellent performance with accuracy of 0.9891 ± 0.0058 and the best Matthews coefficient score, with 0.9637 ± 0.0181, demonstrating effectiveness in identifying true positives and negatives.

On the other hand, CDAC-X also stands out as the best in the Dice metrics, with 0.9680 ± 0.0163, and Jaccard index, with 0.9382 ± 0.0297, reflecting high similarity with the ground truth, although it is surpassed in edge accuracy by methods such as [36] in the Hausdorff distance metric. In terms of convergence time, CDAC-X, with 3.53 s, is relatively efficient, although it is not the fastest.

Despite a higher average time, CDAC-X offers a valuable balance when it comes to performance analysis in validation metrics. The variability in times (standard deviation) of some faster methods can affect the consistency of performance of these models, while CDAC-X maintains an acceptable runtime, justifying its use in contexts that require high quality in segmentation. Therefore, CDAC-X proves its use in contexts that require high quality in segmentation, even if there are faster methods available.

### 3.4. Comorbidity Approach

The performance analysis of lung segmentation in CT images by comorbidity seeks to evaluate CDAC in different clinical conditions, exploring how the method deals with both healthy lungs and those affected by some disease, in the case of this dataset, such as COPD and pulmonary fibrosis. In addition, this analysis helps to identify possible limitations of CDAC, guiding improvements in the algorithm and boosting its reliability and applicability in practice, in a diagnostic aid system.

#### 3.4.1. Analysis Between CDAC Versions—Comorbidity Approach

Table 11 presents the results of the performance metrics for the three CDAC configurations (CDAC-X, CDAC-S and CDAC-A) in lung segmentation using the dataset of chest CT images, adopting a comorbidity approach, which considers the segmentation of a group of healthy lung samples, a group of lung samples with COPD and a group of lung samples with fibrosis, as detailed in Section 2.1. The metrics evaluated were accuracy (Accur), precision (Prec), sensitivity (Sen), specificity (Spe), Matthews correlation coefficient (MCC), Dice similarity coefficient (Dice), Jaccard index (Jac) and Hausdorff distance (HD).

Analyzing Table 11, for healthy lungs, CDAC-X stands out in all metrics, presenting the best results in accuracy (0.9877 ± 0.0055), precision (0.9774 ± 0.0211), sensitivity (0.9762 ± 0.0164), specificity (0.9911 ± 0.0100), MCC (0.9651 ± 0.0083), Dice (0.9734 ± 0.0065), Jaccard (0.9482 ± 0.0124) and Hausdorff (8.2271 ± 1.8225). In lungs with COPD, again, CDAC-X presents the best performance in all metrics. Accuracy is 0.9884 ± 0.0065, precision is 0.9662 ± 0.0278, sensitivity is 0.9710 ± 0.0324, specificity is 0.9932 ± 0.0062, MCC is 0.9575 ± 0.0202, Dice is 0.9641 ± 0.0162, Jaccard is 0.9311 ± 0.0299, and Hausdorff is 4.0302 ± 0.3298. In segmenting lungs with fibrosis, CDAC-X continues to excel with the best accuracy (0.9911 ± 0.0046), precision (0.9644 ± 0.0395), sensitivity (0.9661 ± 0.0133), specificity (0.9959 ± 0.0046), MCC (0.9536 ± 0.0210), Dice (0.9582 ± 0.0193), Jaccard (0.9204 ± 0.0348), and Hausdorff (3.6490 ± 0.2185).

Table 12 shows the average convergence time of the CDAC-X, CDAC-S and CDAC-A versions in the segmentation of lungs in CT images, analyzing healthy lungs, lungs with COPD and lungs with fibrosis. Analyzing this table, it is possible to notice that the CDAC-S version is significantly more efficient in terms of average convergence time for the segmentation of lungs in CT images, regardless of the health condition of the lungs. For healthy lungs, CDAC-S had a convergence time of 3.88 ± 2.96 s, much lower than CDAC-X (7.99 ± 8.57 s) and CDAC-A (7.68 ± 8.92 s). In COPD lungs, CDAC-S was also the fastest, at 2.03 ± 0.94 s, compared with 4.98 ± 5.34 s for CDAC-X and 2.36 ± 1.41 s for CDAC-A. For fibrotic lungs, CDAC-S continued to lead at 1.58 ± 0.808 s, outperforming CDAC-X (7.27 ± 6.27 s) and CDAC-A (5.76 ± 8.43 s).

**Figure 10:**
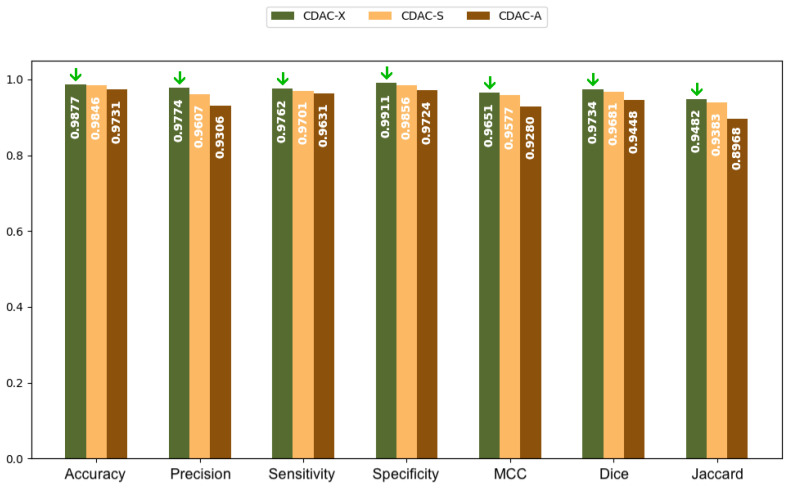
Chart for performance comparison between CDAC configurations, for segmentation of **healthy lungs**, from the metrics presented, in a comorbidity approach.

**Figure 11:**
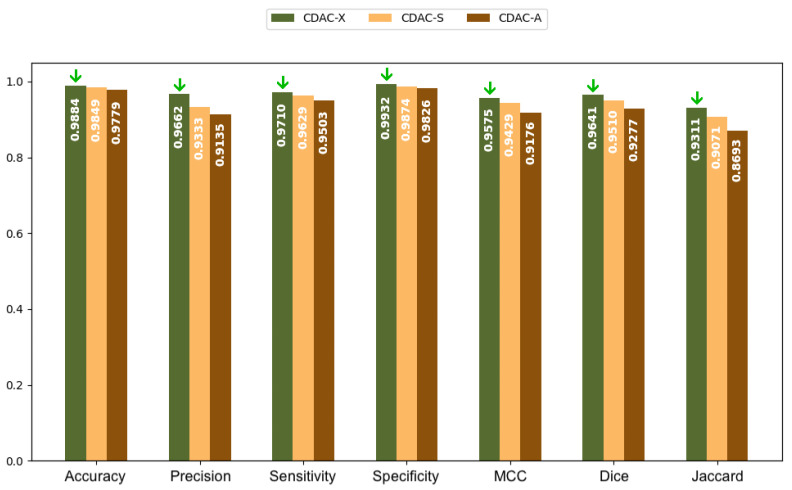
Chart for performance comparison between CDAC configurations, for segmentation of **lungs with COPD**, from the metrics presented, in a comorbidity approach.

**Figure 12:**
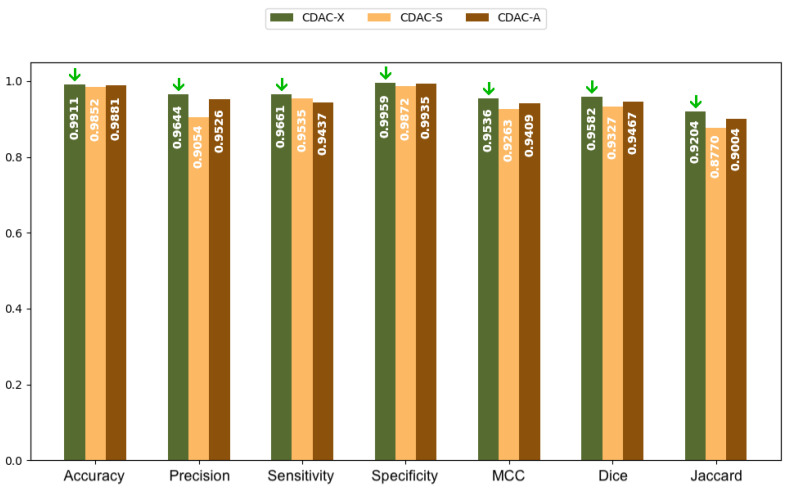
Chart for performance comparison between CDAC configurations, for segmentation of **lungs with fibrosis**, based on the metrics presented, in a comorbidity approach.

In Figure 10, Figure 11 and Figure 12, the bar charts for segmentation of each group of lung samples are shown, being healthy, with COPD and with fibrosis, respectively. The construction of the graphs was based on the values in Table 11. These graphs provide a clearer and more direct comparison of the metrics for the three CDAC configurations in lung segmentation, using images from the chest CT dataset and addressing segmentation by comorbidity. Each CDAC configuration is differentiated by a distinct color, with the legend included in the graph itself. Metrics are presented side-by-side for each configuration, making it easy to see how each one is performing. The best performance for each metric is visually highlighted with a green arrow designating the highest value.

Overall, based on the analysis of Table 11 and Table 12, and consolidated by the graphs shown in Figure 10, Figure 11 and Figure 12, although CDAC-X was not the fastest version, it proved to be the most efficient in terms of overall performance among the versions of the proposed method, considering the comorbidity assessment approach.

The results show that CDAC-X performed best across all metrics and for all lung health conditions. In addition to the performance of the metrics, in the convergence time analysis, CDAC-X was also significantly more efficient, with shorter convergence times across all lung health conditions, with 3.88 s for healthy lungs, 2.03 s for COPD lungs, and 1.58 s for lungs with fibrosis.

#### 3.4.2. Comparison with Other Methods—Comorbidity Approach

Due to its superior performance compared to other versions of CDAC, Table 13 presents a comparison of the results of CDAC-X with other methods, considering metrics such as accuracy, precision, sensitivity, specificity, and Matthews correlation coefficient (MCC). This comparison is also made considering three groups of chest CT image samples with lungs in different health conditions: healthy lungs, lungs with COPD, and lungs with fibrosis.

**Table 13:** Comparison of CDAC-X results with other works for segmentation of the lung dataset—comorbidity approach (part 1).

Methods	Accuracy	Precision	Sensitivity	Specificity	MCC
Healthy Lungs					
**CDAC-X**	0.9877 ± 0.0055	0.9774 ± 0.0211	**0.9762 ± 0.0164**	0.9911 ± 0.0100	**0.9663 ± 0.0087**
He et al. (2023) [32]	0.9845 ± 0.0062	0.9719 ± 0.0225	0.9577 ± 0.0335	0.9889 ± 0.0114	0.9540 ± 0.0162
Filho et al. (2019) [31]	0.9719 ± 0.0082	**0.9917±0.0091**	0.8891 ± 0.0439	**0.9968 ± 0.0038**	0.9200 ± 0.0193
Filho et al. (2014) [12]	**0.9880 ± 0.0057**	0.9777 ± 0.0167	0.9718 ± 0.0080	0.9920 ± 0.0081	0.9651 ± 0.0083
Felix et al. (2013) [33]	0.9453 ± 0.0338	0.8668 ± 0.1061	0.9210 ± 0.0308	0.9476 ± 0.0441	0.8555 ± 0.0667
Rebouças Filho et al. (2011) [34]	0.9731 ± 0.0091	0.9817 ± 0.0222	0.9031 ± 0.0340	0.9936 ± 0.0072	0.9232 ± 0.0192
Alexandria et al. (2010) [35]	0.9631 ± 0.0214	0.9462 ± 0.0529	0.9095 ± 0.0266	0.9770 ± 0.0275	0.9013 ± 0.0373
Felix et al. (2009) [30]	0.9852 ± 0.0060	0.9825 ± 0.0193	0.9543 ± 0.0179	0.9938 ± 0.0075	0.9581 ± 0.0106
Li and Acton (2006) [14]	0.9704 ± 0.0175	0.9721 ± 0.0316	0.9105 ± 0.0317	0.9869 ± 0.0195	0.9197 ± 0.0299
Xu and Prince (1998) [13]	0.9382 ± 0.0489	0.9622 ± 0.0404	0.8410 ± 0.1136	0.9829 ± 0.0211	0.8563 ± 0.0835
COPD Lungs					
**CDAC-X**	0.9884 ± 0.0065	0.9662 ± 0.0278	0.9710 ± 0.0324	**0.9996 ± 0.0010**	**0.9675 ± 0.0202**
He et al. (2023) [32]	0.9814 ± 0.0084	0.9497 ± 0.0327	0.9356 ± 0.0312	0.9903 ± 0.0073	0.9313 ± 0.0242
Filho et al. (2019) [31]	0.9695 ± 0.0060	**0.9981 ± 0.0045**	0.8284 ± 0.0376	0.9932 ± 0.0062	0.8925 ± 0.0215
Filho et al. (2014) [12]	**0.9895 ± 0.0065**	0.9542 ± 0.0325	**0.9801 ± 0.0128**	0.9910 ± 0.0075	0.9607 ± 0.0205
Felix et al. (2013) [33]	0.9794 ± 0.0084	0.9458 ± 0.0534	0.9249 ± 0.0317	0.9887 ± 0.0113	0.9225 ± 0.0251
Rebouças Filho et al. (2011) [34]	0.9827 ± 0.0068	0.9846 ± 0.0227	0.9052 ± 0.0378	0.9968 ± 0.0047	0.9339 ± 0.0248
Alexandria et al. (2010) [35]	0.9812 ± 0.0059	0.9625 ± 0.0418	0.9199 ± 0.0317	0.9921 ± 0.0088	0.9295 ± 0.0162
Felix et al. (2009) [30]	0.9897 ± 0.0039	0.9854 ± 0.0170	0.9481 ± 0.0252	0.9969 ± 0.0036	0.9604 ± 0.0129
Li and Acton (2006) [14]	0.9842 ± 0.0050	0.9834 ± 0.0215	0.9166 ± 0.0370	0.9965 ± 0.0046	0.9400 ± 0.0172
Xu and Prince (1998) [13]	0.9833 ± 0.0061	0.9804 ± 0.0277	0.9144 ± 0.0369	0.9960 ± 0.0060	0.9368 ± 0.0211
Lungs with Fibrosis					
**CDAC-X**	0.9911 ± 0.0046	0.9644 ± 0.0395	0.9661 ± 0.0133	0.9969 ± 0.0046	**0.9576 ± 0.0214**
He et al. (2023) [32]	0.9825 ± 0.0074	0.9481 ± 0.0520	0.8993 ± 0.0411	0.9940 ± 0.0066	0.9129 ± 0.0256
Filho et al. (2019) [31]	0.9780 ± 0.0067	**0.9980 ± 0.0047**	0.8293 ± 0.0298	**0.9998 ± 0.0006**	0.8982 ± 0.0167
Filho et al. (2014) [12]	**0.9920 ± 0.0037**	0.9543 ± 0.0467	**0.9688 ± 0.0172**	0.9950 ± 0.0044	0.9536 ± 0.0210
Felix et al. (2013) [33]	0.9484 ± 0.0516	0.8309 ± 0.1111	0.8085 ± 0.2204	0.9768 ± 0.0164	0.7831 ± 0.1618
Rebouças Filho et al. (2011) [34]	0.9853 ± 0.0049	0.9557 ± 0.0773	0.9090 ± 0.0307	0.9951 ± 0.0069	0.9227 ± 0.0333
Alexandria et al. (2010) [35]	0.9823 ± 0.0075	0.9215 ± 0.0557	0.9183 ± 0.0264	0.9899 ± 0.0070	0.9093 ± 0.0268
Felix et al. (2009) [30]	0.9916 ± 0.0029	0.9768 ± 0.0267	0.9480 ± 0.0244	0.9976 ± 0.0026	0.9574 ± 0.0091
Li and Acton (2006) [14]	0.9817 ± 0.0073	0.9010 ± 0.0777	0.9296 ± 0.0276	0.9876 ± 0.0089	0.9038 ± 0.0338
Xu and Prince (1998) [13]	0.9820 ± 0.0079	0.9299 ± 0.0608	0.9049 ± 0.0365	0.9912 ± 0.0066	0.9066 ± 0.0380

Analyzing Table 13, for the group of samples with healthy lungs, CDAC-X obtained high accuracy (0.9877 ± 0.0055), precision (0.9774 ± 0.0211) and specificity (0.9911 ± 0.0100); however, it stood out for returning the highest sensitivity values, with 0.9762 ± 0.0164, and MCC, with 0.9663 ± 0.0087, surpassing all other methods. For lungs with COPD, CDAC-X maintained its leadership with MCC of 0.9675 ± 0.0202 and achieved the highest Specificity among all, with 0.9996 ± 0.0010. Ref. [12] has a slight advantage in accuracy (0.9895 ± 0.0065) and sensitivity (0.9801 ± 0.0128), while [31] has the highest precision (0.9981 ± 0.0045). For lungs with fibrosis, CDAC-X continues to lead in MCC, with 0.9576 ± 0.0214, and again returns high values in accuracy (0.9911 ± 0.0046), sensitivity (0.9661 ± 0.0133), specificity (0.9969 ± 0.0046), although [12] has the best accuracy (0.9920 ± 0.0037) and sensitivity (0.9688 ± 0.0172) and [31] has the best precision (0.9980 ± 0.0047) and specificity (0.9998 ± 0.0006). The green background in Table 13 highlights instances where CDAC achieved the highest performance in a given metric, ensuring readability with white text. Meanwhile, bold values indicate cases where another method outperformed CDAC in a specific metric.

The best performances of CDAC-X in Table 13 indicate that, for healthy lungs, it presented excellent sensitivity (0.9762 ± 0.0164) and mcc (0.9663 ± 0.0087), showing high efficacy in correctly identifying the areas of the lung to be segmented. For lungs with COPD, it stood out in specificity (0.9996 ± 0.0010) and MCC (0.9675 ± 0.0202), evidencing its accuracy in avoiding false positives and maintaining a balanced segmentation. For lungs with fibrosis, CDAC-X had the best performance in MCC (0.9576 ± 0.0214), reflecting its high quality and balance in segmenting lungs under this specific condition.

In Table 14, the performance of CDAC-X, regarding the analysis by comorbidity, is compared with the same methods, however, using the Dice coefficient, Jaccard index and Hausdorff distance metrics. For healthy lungs, CDAC-X shows the best performance in the Dice, Jaccard and Hausdorff metrics, returning the values of 0.9746 ± 0.0065, 0.9505 ± 0.0124 and 8.2271 ± 1.8225, respectively, significantly outperforming the other methods. In COPD lungs, CDAC-X maintains a satisfactory performance with Dice of 0.9641 ± 0.0162, Jaccard of 0.9311 ± 0.0299 and Hausdorff distance of 11.3717 ± 7.9279; however, it lags slightly behind the method of [12], which presents the best metrics Dice (0.9666 ± 0.0170), Jaccard (0.9359 ± 0.0315), and Hausdorff (3.2058 ± 0.1977). For lungs with fibrosis, CDAC-X again leads with the best Dice (0.9682 ± 0.0193), Jaccard (0.9304 ± 0.0348) and Hausdorff (3.6490 ± 0.2185) metrics, while [31,32,33] show significantly lower values in all metrics.

**Table 14:** Comparison of CDAC-X results with other works for segmentation of the lung dataset—comorbidity approach (part 2).

Methods	Dice	Jaccard	Hausdorff
Healthy Lungs			
**CDAC-X** ^16^	**0.9746 ± 0.0065**	**0.9505 ± 0.0124**	**8.2271 ± 1.8225**
He et al. (2023) [32]	0.9641 ± 0.0158	0.9312 ± 0.0288	39.2823 ± 2.2563
Filho et al. (2019) [31]	0.9383 ± 0.0234	0.8846 ± 0.0408	68.8394 ± 2.5022
Filho et al. (2014) [12]	0.9734 ± 0.0060	0.9482 ± 0.0114	8.3183 ± 1.8190
Felix et al. (2013) [33]	0.8883 ± 0.0488	0.8024 ± 0.0774	23.0173 ± 2.0028
Rebouças Filho et al. (2011) [34]	0.9402 ± 0.0193	0.8878 ± 0.0344	13.2998 ± 1.6877
Alexandria et al. (2010) [35]	0.9261 ± 0.0224	0.8632 ± 0.0383	16.3310 ± 1.6237
Felix et al. (2009) [30]	0.9679 ± 0.0090	0.9380 ± 0.0166	10.9098 ± 1.9122
Li and Acton (2006) [14]	0.9396 ± 0.0184	0.8866 ± 0.0321	14.7047 ± 1.6152
Xu and Prince (1998) [13]	0.8915 ± 0.0619	0.8096 ± 0.0959	19.8571 ± 1.4710
COPD Lungs			
CDAC-X	0.9641 ± 0.0162	0.9311 ± 0.0299	4.0302 ± 0.3298
He et al. (2023) [32]	0.9419 ± 0.0192	0.8908 ± 0.0341	25.2761 ± 1.1491
Filho et al. (2019) [31]	0.9049 ± 0.0221	0.8271 ± 0.0371	78.4890 ± 1.2651
Filho et al. (2014) [12]	**0.9666±0.0170 ^17^**	**0.9359 ± 0.0315**	**3.2058 ± 0.1977**
Felix et al. (2013) [33]	0.9336 ± 0.0202	0.8761 ± 0.0355	10.1916 ± 0.7677
Rebouças Filho et al. (2011) [34]	0.9427 ± 0.0221	0.8923 ± 0.0395	9.0103 ± 0.6018
Alexandria et al. (2010) [35]	0.9395 ± 0.0127	0.8861 ± 0.0223	9.6780 ± 0.6970
Felix et al. (2009) [30]	0.9660 ± 0.0111	0.9345 ± 0.0206	3.7288 ± 0.2011
Li and Acton (2006) [14]	0.9480 ± 0.0157	0.9016 ± 0.0285	7.6722 ± 0.5257
Xu and Prince (1998) [13]	0.9454 ± 0.0186	0.8971 ± 0.0334	7.8677 ± 0.4147
Lungs with Fibrosis			
**CDAC-X**	**0.9682 ± 0.0193**	**0.9304 ± 0.0348**	**3.6490 ± 0.2185**
He et al. (2023) [32]	0.9212 ± 0.0232	0.8547 ± 0.0396	34.5237 ± 1.4902
Filho et al. (2019) [31]	0.9055 ± 0.0168	0.8278 ± 0.0277	84.6300 ± 1.7726
Filho et al. (2014) [12]	0.9605 ± 0.0210	0.9248 ± 0.0377	4.5954 ± 0.7860
Felix et al. (2013) [33]	0.7965 ± 0.1545	0.6858 ± 0.1872	23.9368 ± 2.4316
Rebouças Filho et al. (2011) [34]	0.9289 ± 0.0339	0.8689 ± 0.0547	9.9457 ± 1.4493
Alexandria et al. (2010) [35]	0.9184 ± 0.0232	0.8500 ± 0.0394	13.1974 ± 1.1605
Felix et al. (2009) [30]	0.9616 ± 0.0082	0.9261 ± 0.0151	5.2953 ± 0.6588
Li and Acton (2006) [14]	0.9122 ± 0.0321	0.8402 ± 0.0533	13.4484 ± 1.1233
Xu and Prince (1998) [13]	0.9158 ± 0.0345	0.8464 ± 0.0564	15.1653 ± 1.4811

^16^ The green background highlights instances where CDAC achieved the highest performance in a given metric, with white text ensuring readability. ^17^ Bold values indicate cases where another method outperformed CDAC in a specific metric.

Overall, CDAC-X demonstrates consistent and superior performance in lung segmentation across different health conditions. For healthy lungs, it achieved the best results in sensitivity (0.9762 ± 0.0164) and MCC (0.9663 ± 0.0087), in addition to high accuracy (0.9877 ± 0.0055), high precision (0.9774 ± 0.0211) and high specificity (0.9911 ± 0.0100). In the case of COPD lungs, CDAC-X maintained the lead in MCC (0.9675 ± 0.0202) and specificity (0.9996 ± 0.0010), although [12] showed a slight advantage in accuracy (0.9895 ± 0.0065) and sensitivity (0.9801 ± 0.0128), and [31] in precision (0.9981 ± 0.0045).

For lungs with fibrosis, CDAC-X led in MCC (0.9576 ± 0.0214) and presented high values in accuracy (0.9911 ± 0.0046), sensitivity (0.9661 ± 0.0133) and specificity (0.9969 ± 0.0046), although [12] achieved the best accuracy (0.9920 ± 0.0037) and sensitivity (0.9688 ± 0.0172), and [31] the best precision (0.9980 ± 0.0047) and specificity (0.9998 ± 0.0006).

In metrics such as Dice, Jaccard and Hausdorff, CDAC-X led in healthy lungs with 0.9746 ± 0.0065, 0.9505 ± 0.0124 and 21.1250 ± 38.1209, respectively, and in lungs with fibrosis with 0.9682 ± 0.0193, 0.9304 ± 0.0348 and 10.2843 ± 4.5806. In lungs with COPD, it performed satisfactorily, but slightly worse than [12], highlighting the robustness and efficiency of CDAC-X compared to competing methods. In other words, the values indicate that CDAC-X excels in segmenting healthy and fibrotic lungs, significantly outperforming other methods, and maintains a competitive performance in COPD lungs, although it is not the absolute best in this regard.

**Figure 13:**
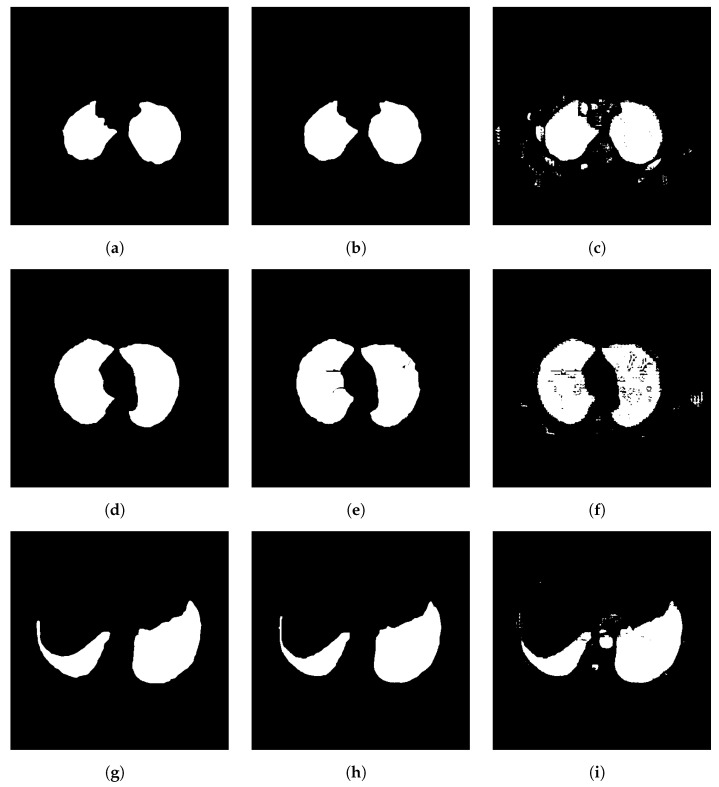
Visual comparison of segmentation results for three different samples: (**a**–**c**) Sample #7 (COPD), (**d**–**f**) Sample #16 (Healthy), and (**g**–**i**) Sample #35 (Fibrosis). Each column presents the ground truth (GT) mask, the result of the proposed CDAC-X method, and the segmentation mask generated by the method used in He et al. (2023) [32]. The comparison highlights the ability of CDAC-X to better align with GT masks across different lung conditions. (**a**) GT Mask—Sample #7 (COPD), (**b**) CDAC-X Mask—Sample #7 (COPD), (**c**) He et al. (2023) [32] mask—Sample #7 (COPD), (**d**) GT mask—Sample #16 (healthy), (**e**) CDAC-X mask—Sample #16 (healthy), (**f**) He et al. (2023) [32] mask—Sample #16 (healthy), (**g**) GT mask—Sample #35 (fibrosis), (**h**) CDAC-X mask—Sample #35 (fibrosis), (**i**) He et al. (2023) [32] mask—Sample #35 (fibrosis).

To further support the quantitative comparisons presented in Table 13 and Table 14, Figure 13 presents a visual comparison of the segmentation results for three different cases: a patient with COPD (Sample #7), a healthy patient (Sample #16), and a patient with fibrosis (Sample #35). The columns display the ground truth (GT) segmentation mask, the result obtained with the proposed CDAC-X method, and the segmentation mask generated by the method used in [32], chosen as an example to perform the visual comparison.

The segmentation results seen in Figure 13 highlight the main differences between the CDAC-X method, the approach proposed in [32], and the ground truth (GT) masks. In the three selected cases—COPD (Sample #7), a healthy lung (Sample #16), and a lung with fibrosis (Sample #35)—CDAC-X consistently demonstrates superior alignment with the GT masks, particularly in regions with irregular boundaries and complex anatomical structures.

In the case of COPD (Sample #7), CDAC-X provides a segmentation that accurately follows the shape and extent of the GT mask, while the method in [32] exhibits slight undersegmentation, failing to fully capture some peripheral regions. This suggests that CDAC-X is more effective in delineating pathological regions where lung structure is altered. For the healthy lung (Sample #16), both CDAC-X and [32] yield segmentations close to the GT mask. However, CDAC-X achieves a more precise contour, while [32] introduces minor deviations, which could impact downstream analyses. This indicates that even in normal anatomical structures, CDAC-X maintains a high level of accuracy.

In the case of fibrosis (Sample #35), the differences become more pronounced. CDAC-X successfully segments lung regions without over-segmentation, preserving the expected anatomical structure. In contrast, the method in [32] shows a tendency towards over-segmentation, including extraneous regions that are not part of the GT mask. This suggests that CDAC-X is better suited to deal with lung conditions with heterogeneous features and diffuse patterns.

Overall, these visual comparisons shown in Figure 13 reinforce the quantitative findings, demonstrating that CDAC-X not only improves segmentation accuracy but also increases robustness across different lung conditions. The method effectively balances sensitivity and specificity, ensuring more reliable segmentation results, particularly in challenging pathological cases.

### 3.5. Computational Cost and Practical Deployment Considerations

Our results indicate that while CDAC-X achieves superior performance in the segmentation task, its computational cost is significantly higher compared to CDAC-S and CDAC-A. This difference can be observed in Table 3, Table 7 and Table 12, which present the computational cost of each variant in individual, general, and comorbidity-based approaches, respectively. The increased complexity of CDAC-X stems from its deeper Vision Transformer (ViT) architecture, as can be seen in Table 1, which enables more sophisticated modeling of anatomical structures and improves segmentation accuracy. However, this performance gain comes at the cost of a substantial increase in computational demand, impacting inference speed and requiring greater processing capacity for efficient execution.

In real-time clinical environments, such as emergency diagnostics and intraoperative navigation, inference speed is a key factor in determining the feasibility of a segmentation method. The higher computational cost of CDAC-X may pose a challenge in these scenarios, especially in systems with limited computing resources. To ensure its viability in such contexts, hardware acceleration techniques such as GPUs, TPUs, or specialized AI accelerators become essential to reduce inference time without compromising model accuracy.

On the other hand, in applications where segmentation accuracy takes precedence over inference speed, such as retrospective analyses, in-depth clinical studies, and scientific investigations, CDAC-X remains a highly relevant option. Its potential to provide more precise and detailed segmentations can be leveraged in medical imaging studies that require high fidelity in anatomical structure identification. Thus, the use of CDAC-X must be balanced between accuracy and computational efficiency, considering the specific requirements of each application. In future work, we plan to explore techniques such as quantization, model compression, and knowledge distillation, aiming to reduce the computational cost of CDAC-X without compromising its accuracy, making it more accessible for real-time applications.

Thus, the use of CDAC must be balanced between accuracy and computational efficiency, considering the specific requirements of each application. In future work, we plan to explore techniques such as quantization, model compression, and knowledge distillation, aiming to reduce the computational cost of CDAC-X without compromising its accuracy, making it more accessible for real-time applications.

### 3.6. CDAC Segmentation Interpretability

One of the main challenges of deep learning-based segmentation methods is their limited interpretability, which often makes it difficult for radiologists to understand the model’s decision-making process. Unlike conventional black-box deep learning approaches, CDAC enhances interpretability by integrating contextual modeling into the segmentation process, allowing for greater user control and transparency.

The contextual attention force (CAF) and contextual balloon force (CBF) explicitly incorporate anatomical and spatial information into the segmentation process, enabling the model to adjust contours dynamically based on clinically relevant contextual cues rather than relying solely on pixel intensity. This approach brings several advantages from an interpretability perspective:

- **Expert-guided initialization:** Radiologists can define an *initial region of interest (ROI)* by marking key areas in the image. This ensures that segmentation aligns with expert knowledge instead of relying exclusively on learned representations;
- **Progressive contour evolution:** The active contour model *gradually adapts its boundaries* based on predefined energy functions (CAF and CBF), allowing for *visual tracking of the segmentation process*, a feature typically absent in deep learning-based approaches;
- **Context-aware decision making:** Instead of solely depending on *global pixel features*, CDAC dynamically adjusts segmentation *based on local image context*, making it easier to interpret why a particular boundary was selected.

These interpretability features make CDAC a promising approach for medical image segmentation, as it allows for greater transparency and user control while maintaining high segmentation accuracy. Future developments will focus on further enhancing interpretability through the integration of explainable AI (XAI) techniques, such as saliency maps and feature attribution methods, to provide additional insights into the model’s decision-making process.

### 3.7. Impact of Hyperparameters on Model Performance

The performance of CDAC is directly influenced by its hyperparameters, particularly the weighting factors in the loss function, which regulate the contribution of internal and external forces to the active contour evolution. Proper adjustment of these parameters contributes to maintaining a balanced trade-off between segmentation accuracy and contour adaptability.

As detailed in Section 2.2, the internal energy term ($E_{CBF}$) consists of a continuity force ($F_{cont}$), which enforces smoothness along the contour, and an adaptive contextual force ($F_{context}$), which adjusts the contour evolution based on local intensity variations and prior contextual knowledge. The external energy term ($E_{CAF}$) represents the contextual attention strength, which modulates the attraction of the contour towards high-relevance regions by leveraging multi-scale semantic features extracted from the Vision Transformer (ViT). The external energy term ($E_{CAF}$) represents the contextual attention force, responsible for guiding the evolution of the contour by integrating contextual features extracted by the Vision Transformer (ViT). This mechanism allows the contour to be attracted to regions of interest based on learned information. This term is not solely based on pixel intensity information but also considers contextual patterns extracted from the image, aiming to contribute to a more stable segmentation that adapts to variations present in medical images.

Fine-tuning these weighting factors is crucial for balancing segmentation accuracy and contour refinement. A higher value for the contextual force weight ($w_{context}$) enhances adaptation to anatomical variations by increasing the influence of high-level contextual information extracted from the Vision Transformer (ViT). However, excessive emphasis on this term may lead to over-segmentation, as the contour becomes overly dependent on global image features rather than local structures. Conversely, increasing the continuity weighting factor ($w_{cont}$) reinforces contour smoothness, which can improve robustness against noise but at the cost of reducing sensitivity to fine anatomical details.

The results indicate that hyperparameter selection significantly impacts segmentation performance, particularly in cases with high anatomical variability. Future works aim to systematically explore and tune these hyperparameters to improve the adaptability of CDAC to other medical imaging modalities, such as magnetic resonance imaging (MRI) and cardiac CT, where anatomical structures and contrast properties differ significantly. Furthermore, additional optimizations will be conducted to refine segmentation performance within the analyzed dataset, ensuring greater robustness across different levels of contrast and anatomical variability.

### 3.8. Clinical Validation and Automated Deployment of CDAC

The segmentation results obtained with CDAC were evaluated against ground truth segmentation masks generated by specialists, ensuring a reliable reference for performance assessment. Both quantitative and qualitative comparisons with these expert-annotated masks demonstrated that CDAC achieves high segmentation accuracy and maintains a strong agreement with reference segmentations, reinforcing its reliability.

The results indicate that CDAC accurately segments pulmonary structures without requiring significant manual adjustments in most cases. Since the model integrates contextual modeling, it effectively adapts contours to anatomical variations without depending on post-processing corrections. However, we acknowledge that in specific cases, such as images with low contrast or severe artifacts, minimal manual adjustments may be required to refine the segmentation output.

Regarding automated deployment, the results suggest that CDAC can be integrated into clinical workflows without frequent manual intervention. Its robustness in segmenting pulmonary structures makes it a strong candidate for real-time applications in medical imaging. However, we recognize that additional validation on diverse datasets is essential for ensuring its generalization to different imaging conditions and equipment settings.

In addition, the computational efficiency of CDAC was analyzed, as discussed in Section 3.5. Although the more complex variant (CDAC-X) achieves higher accuracy, it requires more computational resources, potentially impacting real-time feasibility. Future work should explore hardware acceleration techniques (e.g., GPUs/TPUs) and model compression strategies to optimize inference time while maintaining segmentation performance.

Additionally, we plan to validate CDAC on publicly available datasets, such as LUNA16 [40], to further assess its robustness and adaptability across different clinical scenarios. This validation will be a crucial step in ensuring CDAC’s applicability to a broader range of real-world medical imaging tasks.

## 4. Conclusions

This paper presents the *context-driven active contour* (CDAC), a new model for CT image segmentation that expands the capabilities of active contour models (ACMs) by incorporating contextual information extracted from a *Vision Transformer* (ViT), giving rise to the new driving energies of the ACM concept. Unlike traditional approaches, CDAC not only follows the evolution of the curve, but dynamically adapts its trajectory based on high-level information, ensuring more accurate and robust segmentation. Through initial markings provided by experts, the method integrates the proposed external energy, the *contextual attention force* (CAF), which contextualizes the image from the ViT, with the proposed adaptive internal energy, the *contextual balloon force* (CBF), which intelligently adjusts the stiffness and elasticity of the contour. This process makes segmentation more efficient and reliable, especially for complex medical structures, expanding the potential for application in computer-aided diagnosis.

The main contributions of this work include the introduction of the external energy CAF, which uses contextual information to improve segmentation by reducing noise and improving edge detection, and the creation of the internal energy CBF, which adaptively adjusts the stiffness and elasticity parameters of the active contour according to the image context, optimizing the segmentation process. In addition, the method was evaluated on chest CT images for lung segmentation in different pathological conditions, demonstrating its ability to adapt to anatomical and clinical variations. Finally, the differentiated segmentation by approach allowed individual and global analyses of the lungs, as well as the consideration of comorbidities, providing a comprehensive assessment of the effectiveness of CDAC in various clinical scenarios.

The experimental results with the chest CT dataset demonstrated that the proposed method is capable of convincingly segmenting lung structures, considering different conjunctions. To this end, the analysis of this dataset was conducted considering three distinct approaches: individual analysis, general analysis, and analysis by comorbidity.

In the individual analysis, the lungs were segmented separately, taking into account their specific anatomical and pathological characteristics. The CDAC-X method demonstrated high efficiency in this approach, dynamically adjusting to the structural variations in each lung. In addition, it obtained superior performance metrics than the compared methods, presenting greater accuracy in delimiting the edges and a lower false positive rate.

In the overall analysis, segmentation was performed considering the lungs as a single structure, without distinction between right and left lung. CDAC-X also stood out in this scenario, offering accurate and consistent results, demonstrating its ability to generalize to different lung segmentation contexts. Specifically, this version achieved greater accuracy and sensitivity in detecting lung regions compared to other approaches.

In the comorbidity analysis, segmentation was evaluated in different clinical conditions, such as chronic obstructive pulmonary disease (COPD) and pulmonary fibrosis. CDAC-X demonstrated excellent adaptation to pathological variations, satisfactorily segmenting the regions affected by each disease. In this approach, the model obtained high values of accuracy and specificity, reinforcing its robustness in identifying distinct pathological patterns.

The performance of the newly proposed energies (CAF and CBF) also showed a significant contribution to the success of segmentation with CDAC, avoiding the reanalysis of regions already covered by the contour curve of the proposed model, and demonstrating not only an improvement in the efficiency of the process, but also ensuring greater refinement in the delimitation of the segmented areas.

In summary, the CDAC method, especially in its CDAC-X version, represents a significant advance in medical image segmentation, by incorporating mechanisms and creating new concepts that improve the adaptation and precision of the process. The proposed approach not only improves the efficiency and consistency of segmentation, but also expands the possibilities of applying active contour models in various clinical scenarios, contributing to the evolution of techniques to aid in image diagnosis and driving new research in the area.

### 4.1. Limitations of the CDAC

Based on the analysis of the results presented together with the proposed methodology, possible limitations of the CDAC method include the following:

- **Sensitivity to anatomical variations:** In the analysis of the individual approach, discussed in Section 3.2, CDAC-A exhibited greater sensitivity to the anatomical characteristics of the right lung, leading to less consistent segmentations. This suggests that the method may be influenced by variations in morphology and anatomical structure, such as differences in shape, size, and regional distribution.
- **Computational cost:** Considering the analysis of the general approach, discussed in Section 3.3, while CDAC-S achieves a convergence time of 3.53 seconds, as shown in Table 7, other methods, such as those by Braga et al. [39] (0.13 seconds) and He et al. [32] (1.15 seconds), demonstrate significantly faster processing times, as detailed in Table 10. This highlights the need for optimization in scenarios requiring real-time segmentation.
- **Contour precision:** Although CDAC-X demonstrates high segmentation performance based on Dice and Jaccard metrics, it is outperformed by methods such as de S. Rebouças et al. [36] when evaluated using the Hausdorff distance metric, which measures the precision of segmented contours. This suggests that while CDAC-X is effective in identifying and overlapping regions of interest, it may exhibit inconsistencies or lower accuracy in precisely defining the boundaries of these regions.

Despite these limitations, the CDAC method presents a robust and adaptable approach to lung segmentation, effectively balancing accuracy and contextual awareness. Future research will focus on optimizing computational efficiency, enhancing contour precision, and improving the method’s generalization to a broader range of anatomical variations. These advancements will further strengthen CDAC’s applicability in clinical scenarios.

### 4.2. Future Work

Based on the results obtained and the analyses performed, several directions can be explored in future work to expand and improve CDAC. One of the main challenges to be faced is the reduction in computational cost, which can be made possible through parallelization and optimization of the code, allowing faster convergence of the method, especially in large medical imaging datasets. In addition, validation in different medical imaging modalities is an essential step to assess the versatility of CDAC. The application of the method in magnetic resonance imaging (MRI) and cardiac CT could provide insights into its robustness in different clinical contexts.

CDAC was initially developed and validated using a private chest CT dataset, obtained through a clinical partnership. This dataset allowed for the use of ground truth segmentation masks, annotated by specialists, ensuring a reliable evaluation of the model’s performance. Although the results have demonstrated the effectiveness of CDAC, we recognize the need to evaluate the method on public datasets to validate its generalization under different imaging conditions. As part of our future research, we plan to test CDAC on widely used lung segmentation datasets, such as LUNA16 [40], to enable broader comparisons with other segmentation approaches.

Beyond lung segmentation, CDAC can be applied to other medical imaging modalities, as its structure is based on active contour models (ACMs), guided by contextual information extracted through Vision Transformer (ViT) embeddings. This approach is not restricted to lung CT segmentation and can be adapted for other applications, such as brain MRI, cardiac CT, and abdominal organ segmentation, as discussed at the beginning of this section.

For CDAC adaptation to other anatomical structures, certain modifications would be required:

- Adjustments to the contextual attention force (CAF) and contextual balloon force (CBF) to account for differences in tissue contrast, anatomical variations, and imaging artifacts specific to each modality;
- Training or fine-tuning the model using domain-specific embeddings, optimizing feature extraction relevant to different types of medical imaging.

These adaptations enable CDAC to expand its applicability to a broader range of clinical applications, reinforcing its potential as a versatile segmentation tool based on deep learning and contextual modeling. By allowing adjustments tailored to different imaging modalities, CDAC can be optimized for various medical contexts, including neurological, cardiovascular, and oncological imaging. This flexibility enhances its potential for integration into diverse diagnostic workflows, improving segmentation accuracy and consistency across multiple anatomical structures and imaging techniques.

Another promising prospect is the expansion of CDAC to areas beyond medicine, exploring its use in applications such as agricultural image analysis, surveillance monitoring, and industrial quality inspection, expanding its impact to other sectors. In addition, optimization for different platforms represents a strategic advance, making the method compatible with mobile devices and cloud systems, seeking to promote greater accessibility and effectiveness in different computing environments.

With this in mind, future research will focus on generalizing CDAC, testing its robustness across different medical imaging modalities and non-medical applications, and exploring computational optimization strategies to improve its efficiency and adaptability for a wide range of real-world use cases.

Additionally, we recognize that further improvements in interpretability can be achieved by integrating Explainable AI (XAI) techniques, such as saliency maps, feature attribution methods, or rule-based explanations, to provide additional insights into the decision-making process. As part of our future work, we plan to explore these techniques to enhance CDAC’s transparency and usability in clinical environments, allowing for more reliable and interpretable segmentation results.

Finally, an innovative line of research would be to explore quantum computing to improve the performance of CDAC. The ability of quantum computing to perform large-scale parallel processing could significantly accelerate image segmentation, making the method even more efficient and generalist for different applications. These future directions pave the way for significant advances in the evolution of CDAC, expanding its possibilities for application and improvement.

## Figures and Tables

**Table 1:** Configuration of CDAC-X, CDAC-S and CDAC-A.

Variant	Number of Layers	Heads of Attention	Embedding Size	Parameters (Approx.)
CDAC-S	12	12	768	85 millions
CDAC-A	24	16	1024	307 millions
CDAC-X	32	16	1280	632 millions

**Table 2:** Results for all CDAC configurations with the lung dataset—individual approach.

Metric	CDAC-X ^1^		CDAC-S		CDAC-A	
	**Left Lung**	**Right Lung**	**Left Lung**	**Right Lung**	**Left lung**	**Right Lung**
Accuracy	**0.9951 ± 0.0027**	**0.9940 ± 0.0034**	0.9934 ± 0.0051	0.9915 ± 0.0047	0.9941 ± 0.0030	0.9855 ± 0.0160
Precision	**0.9796 ± 0.0282**	**0.9698 ± 0.0277**	0.9446 ± 0.0617	0.9210 ± 0.0646	0.9728 ± 0.0289	0.8757 ± 0.1620
Sensitivity	**0.9724 ± 0.0235**	**0.9685 ± 0.0236**	0.9655 ± 0.0225	0.9574 ± 0.0312	0.9440 ± 0.0358	0.9610 ± 0.0205
Specificity	**0.9980 ± 0.0029**	**0.9970 ± 0.0033**	0.9947 ± 0.0058	0.9934 ± 0.0049	0.9973 ± 0.0032	0.9872 ± 0.0180
MCC	**0.9662 ± 0.0157**	**0.9600 ± 0.0178**	0.9541 ± 0.0340	0.9393 ± 0.0369	0.9580 ± 0.0175	0.9055 ± 0.0948
Dice	**0.9686 ± 0.0151**	**0.9629 ± 0.0169**	0.9568 ± 0.0346	0.9428 ± 0.0367	0.9606 ± 0.0173	0.9066 ± 0.1000
Jaccard	**0.9396 ± 0.0279**	**0.9290 ± 0.0308**	0.9191 ± 0.0576	0.8939 ± 0.0621	0.9248 ± 0.0317	0.8427 ± 0.1481
Hausdorff	**4.3793 ± 1.1334**	**6.6373 ± 2.6732**	4.8570 ± 1.1162	7.4921 ± 2.6594	5.6548 ± 1.1430	9.0498 ± 2.6279

^1^ The green background highlights instances where CDAC achieved the highest performance in a given metric, with white text ensuring readability.

**Table 3:** Average convergence time for CDAC-X, CDAC-S and CDAC-A versions in the segmentation of lungs in CT images—individual approach.

CDAC Version	Left Lung—Time (s)	Right Lung—Team (s)
CDAC-X	4.42 ± 4.91	6.29 ± 5.55
**CDAC-S ^2^**	**2.32 ± 1.21**	**1.86 ± 0.75**
CDAC-A	2.37 ± 1.25	5.38 ± 6.45

^2^ The green background highlights instances where CDAC performed best, with white text ensuring readability.

**Table 6:** Results for all CDAC configurations with the lung dataset—general approach.

Metric	CDAC-X ^7^	CDAC-S	CDAC-A
Accuracy	**0.9891 ± 0.0058**	0.9849 ± 0.0090	0.9797 ± 0.0172
Precision	**0.9694 ± 0.0310**	0.9332 ± 0.0567	0.9322 ± 0.0800
Sensitivity	**0.9711 ± 0.0216**	0.9622 ± 0.0236	0.9524 ± 0.0251
Specificity	**0.9934 ± 0.0075**	0.9867 ± 0.0111	0.9828 ± 0.0236
MCC	**0.9587 ± 0.0181**	0.9423 ± 0.0337	0.9288 ± 0.0466
Dice	**0.9652 ± 0.0163**	0.9506 ± 0.0317	0.9397 ± 0.0399
Jaccard	**0.9332 ± 0.0297**	0.9075 ± 0.0539	0.8888 ± 0.0676
Hausdorff	**5.2922 ± 1.1140**	5.7942 ± 1.0968	8.2371 ± 1.1682

^7^ The green background highlights instances where CDAC achieved the highest performance in a given metric, with white text ensuring readability.

**Table 7:** Average convergence time for CDAC-X, CDAC-S and CDAC-A versions in the segmentation of lungs in CT images—general approach.

CDAC Version	Time (s)
CDAC-X	7.42 ± 5.24
**CDAC-S** ^8^	**3.53 ± 1.00**
CDAC-A	5.62 ± 4.64

^8^ The green background highlights instances where CDAC performed best, with white text ensuring readability.

**Table 11:** Results for all CDAC configurations with the lung dataset—comorbidity approach.

Metric	CDAC-X ^14^	CDAC-S	CDAC-A
Healthy Lungs			
Accuracy	**0.9877 ± 0.0055**	0.9846 ± 0.0079	0.9731 ± 0.0206
Precision	**0.9774 ± 0.0211**	0.9607 ± 0.0239	0.9306 ± 0.0640
Sensitivity	**0.9762 ± 0.0164**	0.9701 ± 0.0106	0.9631 ± 0.0257
Specificity	**0.9911 ± 0.0100**	0.9856 ± 0.0121	0.9724 ± 0.0306
MCC	**0.9651 ± 0.0083**	0.9577 ± 0.0132	0.9280 ± 0.0411
Dice	**0.9734 ± 0.0065**	0.9681 ± 0.0092	0.9448 ± 0.0295
Jaccard	**0.9482 ± 0.0124**	0.9383 ± 0.0170	0.8968 ± 0.0516
Hausdorff	**8.2271 ± 1.8225**	8.2273 ± 1.8031	12.7296 ± 1.7132
COPD Lungs			
Accuracy	**0.9884 ± 0.0065**	0.9849 ± 0.0067	0.9779 ± 0.0164
Precision	**0.9662 ± 0.0278**	0.9333 ± 0.0345	0.9135 ± 0.1017
Sensitivity	**0.9710 ± 0.0324**	0.9629 ± 0.0262	0.9503 ± 0.0242
Specificity	**0.9932 ± 0.0062**	0.9874 ± 0.0080	0.9826 ± 0.0205
MCC	**0.9575 ± 0.0202**	0.9429 ± 0.0218	0.9176 ± 0.0573
Dice	**0.9641 ± 0.0162**	0.9510 ± 0.0184	0.9277 ± 0.0514
Jaccard	**0.9311 ± 0.0299**	0.9071 ± 0.0331	0.8693 ± 0.0867
Hausdorff	**4.0302 ± 0.3298**	4.0421 ± 0.1772	7.6557 ± 0.4858
Lungs with Fibrosis			
Accuracy	**0.9911 ± 0.0046**	0.9852 ± 0.0116	0.9881 ± 0.0090
Precision	**0.9644 ± 0.0395**	0.9054 ± 0.0798	0.9526 ± 0.0631
Sensitivity	**0.9661 ± 0.0133**	0.9535 ± 0.0242	0.9437 ± 0.0210
Specificity	**0.9959 ± 0.0046**	0.9872 ± 0.0125	0.9935 ± 0.0098
MCC	**0.9536 ± 0.0210**	0.9263 ± 0.0477	0.9409 ± 0.0358
Dice	**0.9582 ± 0.0193**	0.9327 ± 0.0443	0.9467 ± 0.0323
Jaccard	**0.9204 ± 0.0348**	0.8770 ± 0.0738	0.9004 ± 0.0543
Hausdorff	**3.6490 ± 0.2185**	5.1302 ± 0.4021	4.2861 ± 0.3774

^14^ The green background highlights instances where CDAC achieved the highest performance in a given metric, with white text ensuring readability.

**Table 12:** Mean convergence time for CDAC-X, CDAC-S and CDAC-A versions in the segmentation of lungs in CT images—comorbidity approach.

CDAC Version	Healthy Lung—Time (s)	COPD Lung—Time (s)	Lung with Fibrosis—Time (s)
CDAC-X	7.99 ± 8.57	4.98 ± 5.34	7.27 ± 6.27
**CDAC-S** ^15^	**3.88 ± 2.96**	**2.03 ± 0.94**	**1.58 ± 0.808**
CDAC-A	7.68 ± 8.92	2.36 ± 1.41	5.76 ± 8.43

^15^ The green background highlights instances where CDAC performed best, with white text ensuring readability.

## Data Availability

Data underlying this paper will be shared on reasonable request to the corresponding author Pedro Pedrosa Rebouças Filho.
